# A Machine Learning Pipeline for Predicting Pinot Noir Wine Quality from Viticulture Data: Development and Implementation

**DOI:** 10.3390/foods13193091

**Published:** 2024-09-27

**Authors:** Don Kulasiri, Sarawoot Somin, Samantha Kumara Pathirannahalage

**Affiliations:** Centre for Advanced Computational Solutions (C-fACS), Lincoln University, Lincoln 7647, New Zealand

**Keywords:** machine learning, pipeline, Pinot Noir, grapes, viticulture, yield, wine quality

## Abstract

The quality of wine depends upon the quality of the grapes, which, in turn, are affected by different viticulture aspects and the climate during the grape-growing season. Obtaining wine professionals’ judgments of the intrinsic qualities of selected wine products is a time-consuming task. It is also expensive. Instead of waiting for the wine to be produced, it is better to have an idea of the quality before harvesting, so that wine growers and wine manufacturers can use high-quality grapes. The main aim of the present study was to investigate the use of machine learning aspects in predicting Pinot Noir wine quality and to develop a pipeline which represents the major steps from vineyards to wine quality indices. This study is specifically related to Pinot Noir wines based on experiments conducted in vineyards and grapes produced from those vineyards. Climate factors and other wine production factors affect the wine quality, but our emphasis was to relate viticulture parameters to grape composition and then relate the chemical composition to quality as measured by the experts. This pipeline outputs the predicted yield, values for basic parameters of grape juice composition, values for basic parameters of the wine composition, and quality. We also found that the yield could be predicted because of input data related to the characteristics of the vineyards. Finally, through the creation of a web-based application, we investigated the balance of berry yield and wine quality. Using these tools further developed, vineyard owners should be able to predict the quality of the wine they intend to produce from their vineyards before the grapes are even harvested.

## 1. Introduction and Background

Wine is either whole or partially fermented fresh grapes or grape juice with an alcohol content of no less than 8.5% (volume). While it has been part of different cultures all over the world for thousands of years, today wine drinking is particularly popular in Western countries. The global consumption of wine is significant, with consumers drinking more than 225 hectolitres each year for the previous two decades.

Current concerns about the quality of wine products have arisen among wine consumers and the wine manufacturing industries. Competition between vineyards to increase their sales by marketing acquired quality certificates has become more widespread in recent times. Today, winemakers are increasingly adopting new technologies, both in the field of viticulture and the winemaking process, to increase the quality of their wine products. It is also important for winemakers to be able to test the quality of their products as this helps them with the marketing of their products. However, the procedure of testing the product’s quality at the end of the production line is time-consuming and expensive because it relies on the services of professional wine tasters.

By knowing the yield of their vineyards in advance, wine growers and wine manufacturers can maintain the best balance between vegetative and reproductive growth. This information is also helpful when making decisions related to thinning, irrigation, nutrient management, scheduling harvests, optimising winemaking operations, programming crop insurance, and determining how many staff will be needed at harvest time. The traditional methods used to predict a vineyard’s yield are time-consuming and labour-intensive. As a result, this has become a hot topic in viticulture research around the globe. This paper proposes a web-based application to predict the quality of wine products based on viticulture parameters. We focus specifically on Pinot Noir wines produced by New Zealand manufacturers using grapes grown in local vineyards. In our study, we investigated the ability to use machine learning to analyse a dataset related to viticulture, concentrating on vineyard yield and the quality of the wine product.

### 1.1. Pinot Noir Wines

Andrew Barr defined Pinot Noir as the grape of Burgundy, known as the finest wine in the world [1]. However, unless the right clone of Pinot is grown using the right viticulture techniques or plant training system in exactly the right climate and picked at precisely the right time, the wine will not meet these quality standards. In terms of the total number of hectares grown, Pinot Noir is the most grown grape variety in New Zealand.

Climate control is crucial for maintaining the quality of Pinot Noir wines. If the temperature is too hot, the fruit may be overripened and mushy. In contrast, in extremely cold weather, the fruit tastes sour and has little flavour. These temperature demands mean that the variety is best suited to cooler climates. To produce high-quality products, managers must also consider soil and vineyard management techniques such as vine spacing, yields, fertilisation, rootstock, clones, and the actual winemaking procedure. In comparison to other grape harvests, the yield for Pinot Noir is moderately low.

Pinot Noir wines are full-bodied, soft, and delicate. They have an intense, bright ruby-red colour. Pinot Noir typically smells like sweet fruit; they can contain cherry, blackberry, strawberry, plum, and blackcurrant flavours, with hints of almonds and flowers like violets. The aroma of Pinot Noir wines may be like fresh strawberries, wild berries, cherries, or plums.

The chemical composition of the wine governs the wine’s major characteristics such as its flavour, fragrance, and colour [2,3]. While anthocyanins are the major contributors to the colour of red wine, tannins contribute to wine astringency. Volatile phenols, alcohols, and norisoprenoids are crucial to the aroma of wine products. Volatile sulphur compounds have a strong connection to sensory feelings. All these important components of Pinot Noir wines have a strong effect on the quality of the product. Pinot Noir grapes generally feature lower anthocyanin concentrations and higher tannin concentrations.

### 1.2. Wine Quality

The quality of any wine depends on the grape composition, which is correlated with geological and soil variables, climate, and viticultural decisions [4]. Oenological practices also affect wine quality. According to prior research, the quality of red wines depends on the qualitative and quantitative composition of the aromatic compounds having various chemical structures and properties and their interactions within different red wine matrices [5]. Certain viticultural regions are known for producing high-quality fruit, which results in better wine. This fact explains the different retail prices for the same type of wine [6]. Climatic factors affect the ripening dates, the composition of several compounds (including 1-hexanol and the monoterpenoids linalool and α-terpineol in grape juice), and alcohol levels in the wine [7].

However, it is difficult to determine wine quality since it is subjective and depends on an individual’s perception. Charters and Pettigrew found that perceptions of wine quality differ among different populations [8]. To identify a wine’s quality, consumers often read wine experts’ reviews and consider other information such as price, geographical origin, and the age of the wine product [9].

However, for many consumers, wine quality is subjective, regardless of whether they are experts or not. Experts offer a unique viewpoint on wine quality due to their deep understanding of wine production, particularly the chemical composition of wine. Non-experts, on the other hand, are more likely to describe wine quality in terms of price, presentation, and provenance. The flavour, fragrance, colour, and other characteristics are determined by the chemical composition of wine [10], which is related to the grape type, environment, microbial strains present during fermentation, and viticulture practices. Volatile compounds are responsible for wine’s fragrance, and phenolic compounds give it its flavour. Laboratory tests determine wine characteristics such as pH, alcohol content, total sulphur, and anthocyanin levels, which are all important in wine quality certification. The percentage of alcohol in wine products has a significant effect on the perception of quality since this is strongly correlated with flavour and aromas [11]. Alcohol aids in the release of volatile aromatic compounds. It is worth mentioning that the contribution of aroma compounds to wine quality is not defined by their concentrations, because some compounds can participate highly in wine aroma even at lower concentrations due to high odour thresholds. Due to climatic changes, producing high-quality and reasonably priced Pinot Noir has become challenging for New Zealand winegrowers and wine manufacturers. There is evidence that phenolic compounds drive quality. Wine quality assessment is a holistic exercise based on aroma, colour, taste balance, and aroma, along with other heuristic attributes.

### 1.3. Machine Learning in Viticulture

Machine Learning (ML) is a powerful predictive tool. The goal is to construct computer programmes that can learn by themselves using a particular set of data. ML uses several types of algorithms to analyse a particular dataset or to make predictions. Classification and regression predict the value of one field (the target) based on the values of the other fields (attributes or features). If the target is discrete (e.g., nominal or ordinal) then the given task is called classification; if the target is continuous, the task is called regression. Classification or regression are typically supervised procedures: using a previously correctly labelled set of training instances, the model learns to correctly label new unseen instances. When the algorithm is tested on unlabelled data it will predict an unknown value as one of the labels it was trained with. Clustering is an unsupervised task whose aim is to group a set of objects into classes of similar objects. A cluster is a collection of similar objects: they differ from the objects in other clusters. The most important notion in clustering is the notion of similarity.

Forecasting grape yield is crucial for the wine industry. Having accurate forecasting helps managers to make decisions related to investments like equipment, the pricing of products, scheduling labour, and developing marketing strategies. Most of the models use shape detection with colour information or a semi-supervised Support Vector Machine (SVM) classifier or k-nearest neighbour classifier. These techniques can be used to detect grape bunches, determine the size and weight of the grapes, and estimate the yield [12,13,14,15,16,17,18,19]. Detecting disease is another critical aspect of viticulture as diseases can cause severe economic losses. These diseases are caused either by fungi or bacteria. Common grape diseases include downy mildew, powdery mildew, anthracnose, grey mould, and black rot. Hence, it is crucial to detect any diseases in the vineyard as early as possible. The current research in viticulture uses image processing, computer vision, and machine learning to detect diseases in grape leaves and fruits. Grapevine pruning results in better grape formation, maintains vine form, improves the quality of the grapes and the resulting wine, and stabilises production over time. The detection of buds in winter is important for grapevine pruning and grapevine plant phenotyping. Bud detection models use SVM to detect grapevine buds in winter [20]. Bunch compactness is another critical issue in viticulture because it may affect berry size, yield, and fruit split. It is also important for ensuring that the fruit ripens at the same time and reducing the incidence of disease. A combination of different machine learning and computer vision techniques could be used to determine the compactness of the acquired images [21]. Seed maturity is used as an indicator of ripeness. Managers need this information to decide when the best time to harvest the fruit is; this ensures the production of top-quality wine. One study used a hybrid segmentation technique to classify seeds according to their degree of maturity [22]. Machine learning models for the estimation of grape ripeness and seed maturity have been developed using SVM, Multiple Linear Regressor, and neural networks [23]. These machine learning techniques, along with image processing and computer vision techniques, can be applied in smart vineyards, vineyard management, and winemaking processes. Future vineyards may use fast and efficient data provided by vehicle-mounted camera systems. Such technology would enable managers to make faster decisions when dealing with critical problems such as plant diseases. It would also help them to decide when the best time is to harvest the fruit.

Machine Learning has recently been used to predict the quality of wine products. Several studies have attempted to identify essential features that affect wine quality and to predict wine quality using a variety of machine learning methods, especially in red wines [24,25]. They have also compared different classification algorithms such as the SVM model, random forest, and Nave Bayes algorithms, decision tree classifier, and the k-nearest neighbour algorithm [26,27,28,29,30]. For example, Piyush et al. examined chemical (47 features) and physicochemical (7 features) data from New Zealand Pinot Noir and compared machine learning algorithms to predict the quality of wine products [10]. Another study developed an integrative machine learning tool based on near-infrared spectroscopy (NIR) from Pinot Noir wines from a vertical vintage. It examined the effects of seasonal weather patterns and water management practices to assess the sensory profiles of wines before the final wine was produced [31]. They used weather data and management practices to predict the colour of the wine [31]. In another study, Fuentes et al. proposed a set of machine learning models that winemakers can use to assess the aroma profiles of wines before beginning the winemaking process. This tool could help wine growers and manufacturers to maintain or increase the quality of their wines or produce wine styles that reflect their specific vineyards or the region where they are located [32].

## 2. A Vine-to-Wine Quality Pipeline

**Figure 1:**
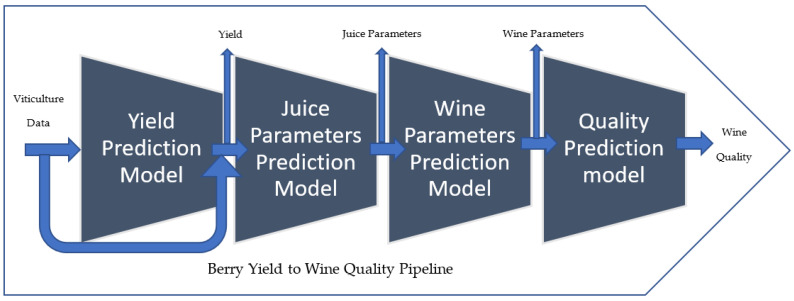
Viticulture-to-wine quality pipeline. This model takes viticulture data as inputs and predicts the yield with regard to the input. The second step predicts selected sets of chemical compositions measured in juice analysis. The juice parameters were taken as the inputs for the third step of the pipeline, and chemical substances measured during wine analysis were predicted. The last step predicts the quality of the wine product using wine composition as the input.

The viticulture-to-wine quality pipeline is a series of steps that wine growers or manufacturers can use to predict the quality of the wine product from viticulture-related features. Figure 1 below describes the pipeline proposed for this.

The winemaking process begins in the vineyards. A high-quality wine relies on high-quality grapes. When the grapes are ripened, there must be a balance between the sugar and acidity levels. Wine growers then harvest the yield and transport the fruit to the winery (STEP 1). Grapes are sorted to identify healthy clean grapes, with the chosen ones crushed into grape juice (STEP 2). The wine-making process entails fermentation, racking, clarification, filtration, maturation, bottling, and ageing (STEP 3). The wine is tested by wine reviewers to measure the quality of the final output (STEP 4). Winemaking is a delicate science. Traditional techniques are combined with modern technology to press, ferment, and mature the delicate grapes/fruits into the world’s most popular drink.

The pipeline includes four steps, each of which is linked to the four steps of the winemaking process and a machine learning model. Whereas the first model predicts the yield of vineyards from the viticulture dataset as inputs, the second predicts the number of selected chemicals in the grape juice. The third model predicts a selected set of wine chemicals. The final model takes the wine parameters as input and predicts the quality of the wine produced (output).

**Figure 2:**
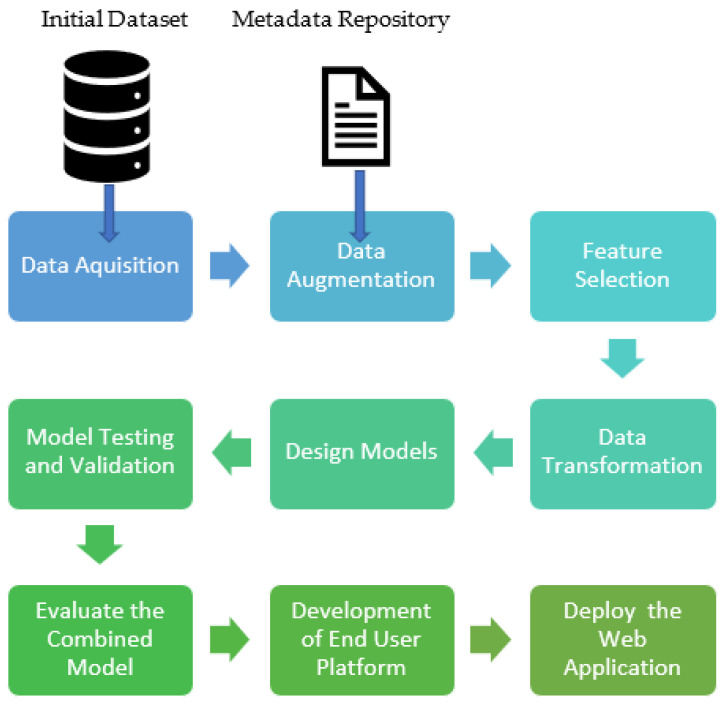
Machine learning pipeline for the model: from data acquisition to development of end-user application.

The machine learning pipeline of the proposed model includes multiple sequential steps that do everything from data extraction and data pre-processing to model training and deployment. Figure 2 provides a schematic diagram of the machine learning pipeline process we followed throughout our research. Each step in the pipeline is discussed in further detail in later sections (Section 3.1, Section 3.2 and Section 3.3).

### 2.1. Data Acquisition

Most of the Pinot Noir vineyards in New Zealand are located in regions of the South Island that have dry climatic conditions and cool nights [33,34]. These conditions preserve the acidity and other characteristics related to the wine flavour [35]. In this study, data were collected from 12 vineyards situated in Central Otago, Marlborough, and Wairarapa, regions with similar climatic conditions. The chosen vineyards are well known for producing high-quality Pinot Noir. The 12 commercial vineyards comprise eight single-vineyard “icon” wines and four multi-vineyard blends or “affordable” wines. While the average price of “icon” wines is approximately NZD 75, those in the affordable wine group have an average price of NZD 24 [10]. Data collection and analysis occurred in 2018, 2019, and 2021. As part of a larger research program to examine links between composition and quality in New Zealand Pinot Noir, 18 wines were selected to be representative of current production practices [10]. The wines chosen were from different producers from different regions within New Zealand: Nelson, North Canterbury, Wairarapa, Central Otago, and Marlborough. Of the 18 bottles, 16 were from the 2016 vintage; the remaining two were from 2013. Six bottles involved in this study were considered to be of commercial quality, while the remaining 12 bottles were considered premium. Sixteen of the bottles had screw caps and the remaining two had corks. Grape harvesting for the premium wines was conducted by hand, a process which generally results in a much lower yield. Grapes for commercial use were harvested using machines, resulting in moderate to high yields. Conventional forms of viticulture were used for the commercial wines; in contrast, the premium wines used an organic approach (except for the premium wine from the Craggy range). The Pinot Noir bottles mentioned are assigned a number from 1 to 18 for further study of these data. Forty-seven aroma compounds previously identified in Pinot Noir were analysed by headspace–SPME gas chromatography–mass spectrometry (HS-SPME GC-MS). The details of the quality assessment of these wines are given in [36].

An extensive publication of the viticulture research within this research program has been published by Damian Martin et al. [37], and the ML research presented in this paper is based on their raw data, which were given in [37] in 2020. Very briefly, to summarise what Martin et al. [37] wrote, the following quantities were measured: the total number of shoots, the number of shoots greater than 5 mm in size, the number of shoots less than 5 mm in size, the number of blind buds, the percentage leaf area in the fruit zone, the percentage of vine canopy, the leaf area per vine, the leaf area per metre, the mean berry weight, the total yield per metre, the total yield per one square metre, and total yield per vine. A total of 50 fresh grape samples were used to calculate the mean berry weight.

Grapes were crushed by hand in a plastic sample bag for the grape juice analysis. During juice analysis, we measured δ13CVPDB (the result of analysis on carbon isotypes), total soluble solids, pH value, titratable acidity, primary amino acids, malic acid, tartaric acid, ammonium, calcium, magnesium, potassium, alanine, arginine, aspartic acid, glutamic acid, serine, threonine, and tyrosine. In addition, we measured the mean optical densities (ODs) of the berry extracts at three different wavelengths: 280, 320, and 520 nm.

**Figure 3:**
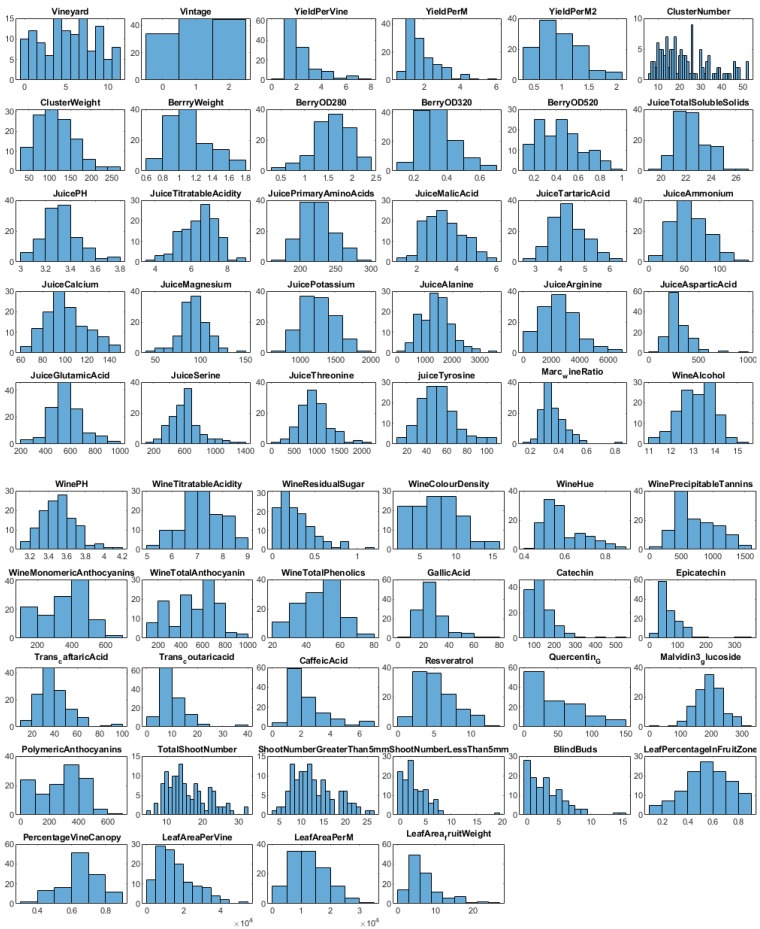
Histogram of all the features in the original dataset.

We also obtained Marc measurements: wine ratio, alcohol, pH value, titratable acidity, residual sugar, colour density, hue, methyl cellulose precipitable tannins, monomeric anthocyanins, total phenolics, gallic acid, catechin, epicatechin, trans-caftaric acid, trans-coumaric acid, caffeic acid, resveratrol, Quercetin-G, malvidin 3-glucoside, and polymeric anthocyanins levels. The SHAP value analysis and Figure 3 provide a histogram of the 58 features collected during the viticulture, juice, and wine analyses.

### 2.2. SHAP Value Analysis

In scientific scenarios, both the designer and the end user may be curious about why the model predicted a certain value for a selected sample. For instance, in a drug effectiveness prediction model, the end user may want to know why s/he obtained a certain effectiveness value. Interpretability is vital for increasing the social acceptance of these models [38]. Shapley values can be used to explain the output of a machine learning model. This technique shows how much of an impact a certain feature has on the final prediction.

**Figure 4:**
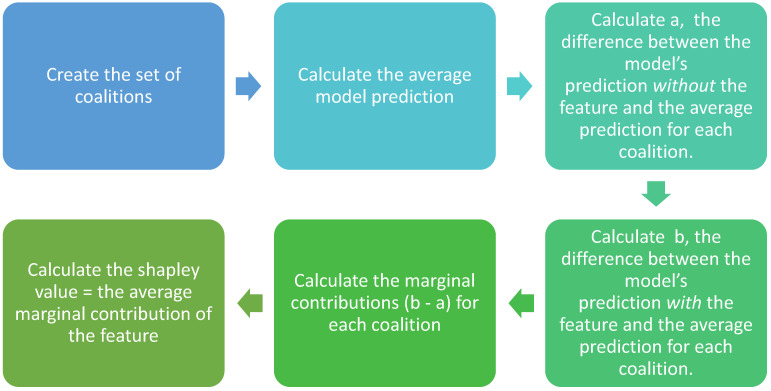
Step-by-step approach for calculating the Shapley value.

The model calculates the Shapley value of a feature following a step-by-step approach (Figure 4). First, it considers the whole set of possible combinations of the input features. These combinations are referred to as coalitions. Second, it calculates the average model prediction. Third, it calculates the difference between the model’s prediction without the selected feature for each coalition and the average prediction. Fourth, it calculates the difference between the model’s prediction, with the selected feature and the average prediction. Fifth, it determines the impact of the feature on the model’s prediction from the average. This step calculates the difference between the resulting values in the third and fourth steps. The resulting value is the marginal contribution of the selected feature. Finally, the Shapley value is calculated using the average of the feature’s marginal contributions [39].

Once the Shapley values for all features have been calculated, we can obtain the global interpretation in a combined form using a summary plot. Here, Shapley values are positioned on the *x*-axis, with features given on the *y*-axis.

**Figure 5:**
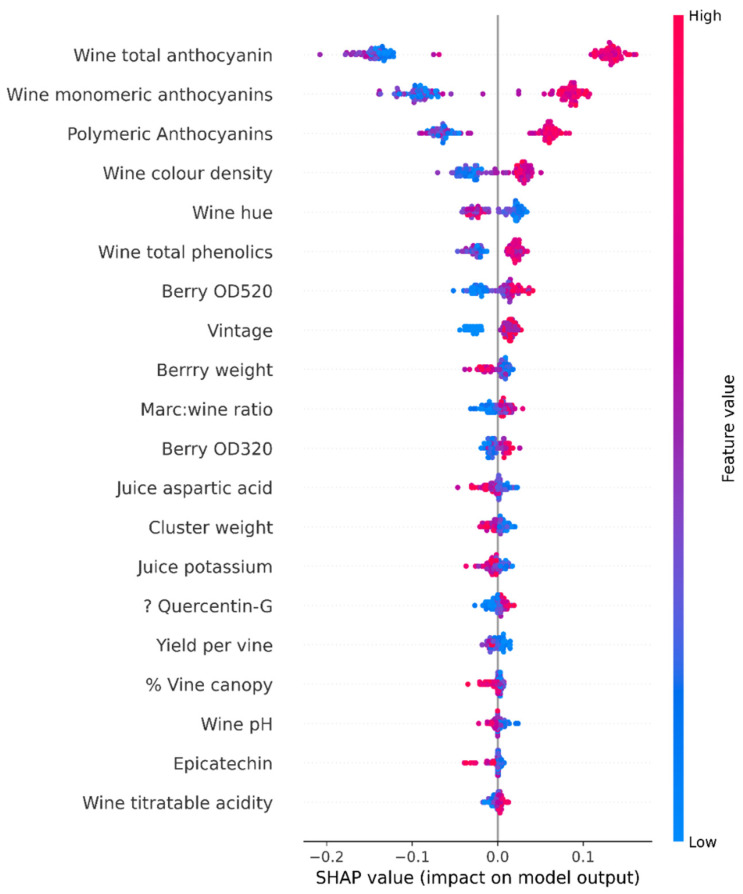
SHAP value diagram for quality.

In terms of explainability, Shapley values provide a full explanation of the model’s features [38]. However, there are problems with this approach. This process requires a lot of computing time. For example, for n set of features of a dataset, there can be 2^n^ possible coalitions of the subsets of features. Missing values are filled with random values. This practice may affect the Shapley value estimations. Figure 5 shows the SHAP value summary plot for quality based on other parameters as an example.

### 2.3. R2 Scores in Linear Regression

The R2 score, which is known as the coefficient of determination, is one of the most important metrics when evaluating regression models with continuous targets. This technique calculates the square of the correlation between two datasets. The R2 score provides an indication of a model’s goodness of fit. For example, an R2 score lies between 0 (no correlation) and 1 (strong correlation): the closer it is to 1, the better the regression fit [40]. A low R2 score is generally a bad sign for predictive models; however, in some cases, a good model may have a small value. We used the following equation to calculate the R2 score.

$$R2=\frac{SSR}{SST}=\frac{regression\;sum\;of\;squares}{total\;sum\;of\;squares}$$

The R2 score is the most common interpretation of how well the regression model explains the observed data. For instance, if the model has an R2 score of 90%, this indicates that there is 90% variability in the target variable in the regression model.

### 2.4. Feature Extraction

Different algorithms were tested to select features or reduce dimensionality when defining the wine quality. We chose the SelectKBest method, which selects features according to the k highest score by calculating the *p* values and important scores for each feature against the output (quality/yield). Of the number of score functions within SelectKBest, including f_regression, mutual_info_regression, chi2, f_classif, and mutual_info_classif, f_regression and mutual_info_regression were used in our analysis since they are specially designed for regression analysis [41]. This process removes all the unimportant features from the dataset, except the k number of features with the highest scores. Feature selection reduces overfitting by preventing the models from making decisions based on redundant data/noise. It also improves the accuracy of the models by removing misleading data. Reducing training time is another noteworthy advantage of feature selection because removing unimportant features reduces the size of the dataset significantly. However, wine quality is a subjective exercise, so any feature selection methods are limited to the datasets that have been used. Therefore, the features that are selected must be checked against expert judgments and historical knowledge among winemakers.

#### 2.4.1. Feature Selection for the Models

Data pre-processing is a significant step in the machine learning approach and is used to transform the raw data into a useful and efficient format. This includes feature extraction, correlation matrix, and data transformation for a better experience during data analysis. We included 58 features given in Figure 1 in our dataset. They represent different stages of manufacturing, beginning from viticulture and ending with the finished product.

First, we divided the features into four steps that represent the four models of the pipeline: features related to yield, features related to juice analysis, features related to wine analysis, and features related to the quality of the chosen wine products. We then performed SHAP value analysis and feature extraction to identify the most important features for the four models/stages based on the SHAP values, which represent the impact of the features on the selected output.

As the SHAP value results in Section S1 (See Supplementary Material Figures S1–S45) show, the first model identified four input features and three output features (Figure S9). The second model has six input features and 14 output features (Figure S10). The third model has 14 input features and five output features (Figure S11). The final model has five input features and one output feature (quality) (Figure S12).

#### 2.4.2. Data Augmentation

If the dataset consists of a smaller number of samples, then synthetic data augmentation is an important step. Our dataset contained 123 samples of data which were not enough to train the model when the dataset was divided for training, testing, and validation. Data augmentation either increases the amount of data by adding modified clones of the current dataset or creating synthetic data from the existing dataset/s. There are many data augmentation techniques that can be used to produce a rich and sufficient set of synthetic data and ensure that the model performs better and has greater accuracy.

We used the synthetic data vault (SDV) package [42] with FAST_ML preset [43] that applies machine learning to models and generates synthetic data using 6000 samples. This process captures the correlations that exist between the features of the original dataset and uses basic statistical properties, such as min/max values, averages, and standard deviations of the features of the original dataset, to generate high-quality, synthetic data. The modelling step is optimised for speedy data generation.

However, synthetic data augmentation has several issues, including overfitting and imbalanced classification of datasets. To cope with overfitting, we used only 70% of the original data for synthetic data generation. We used the remaining data to test the models. In addition, we used the synthetic minority oversampling technique (SMOTE) package, which synthesises new examples for the minority class to maintain the balance between classes [44].

### 2.5. Data Transformation

Log transformation is one of the most famous transformation techniques that scientists can use to deal with skewed data in biomedical and psychosocial research [45]. The highly non-linear and non-monotonic behaviour of the original dataset of our research led us to find a better way to transform the dataset into another dataset so that non-linear behaviour was reduced. We used log transformation for this purpose since it is believed that log transformation can decrease the variability of data and make data conform more closely to the normal distribution [46]. Log transformation can make patterns more visible. It also reduces the variability of data.

**Figure 6:**
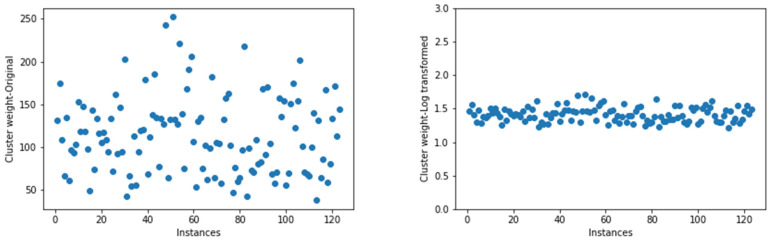
Comparison between the original values and log-transformed values of the feature ‘Cluster Weight (g)’.

For instance, Figure 6 below compares the original values and log-transformed values for the feature ‘cluster weight’. The original values ranged from 8.16 to 252.22. The range for log-transformed values was from 1.22 to 1.71. The figure shows how a log transformation can make patterns more visible.

We used the following formula to calculate the lognormal transformed value *y* from the original value *x*. A and B are constants that vary from one feature to another.

(1) $$y=\ln\left(\left(x\times A\right)+B\right)$$

Log normal transformation is better than min/max normalisation because the variance cannot be reduced using the latter.

Section S2 in the Supplementary Material provides information about the log transformations used for the inputs and exponential transformations for the outputs of the four models.

## 3. Development of Sub-Models

We used neural networks and random forest algorithms in the development of sub-models.

**Figure 7:**
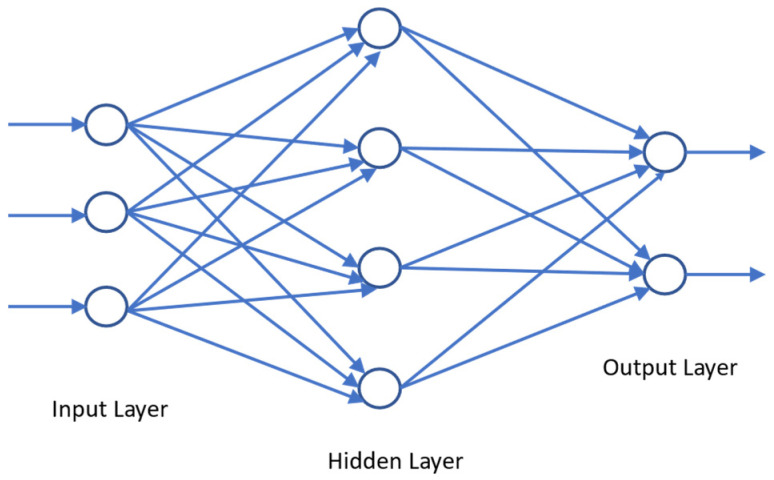
A simple perceptron model with one hidden layer.

Multi-layer perceptron (MLP) is a feed-forward neural network that consists of three types of layers: the input layer, the output layer, and the hidden layer [47]. The data flows in a ‘forwards’ direction from the input to the output layer. Each neuron of each layer is trained with the backpropagation learning algorithm [48]. A simple multilayer perceptron model with one hidden layer is shown in Figure 7. Each layer consists of several neurons whose basic structure resembles the brain’s neurons. The output of a neuron can be expressed as a function of its inputs and weights as is shown in Equation (1) provided below [49].

(2) $$f\left(x,w\right)=x_{1}.w_{1}+x_{2}.w_{2}+\ldots +x_{n}.w_{n}$$

The model is trained continuously in several epochs where the error is backpropagated to modify the weights to increase the accuracy. Neurons of each layer are associated with an activation function [50]. Some of the most popular activation functions for regression are hyperbolic tangent function (tanh), rectified linear unit (ReLU), leaky rectified linear unit (leaky ReLU), and exponential linear unit (ELU) [51].

Additionally, to increase the model’s training efficiency, a user can employ the model’s training efficiency deep learning optimisation algorithms [52]. The goal of model optimisation is to minimise training errors. Some of the commonly used activation functions are stochastic gradient descent (SGD), adaptive gradient (degrade), adaptive moment estimation (adam) and adam with Nesterov momentum (Adam) [53].

The random forest algorithm is one of the most commonly used supervised machine learning algorithms: it is widely used for classification and regression problems. The algorithm is based on decision tree algorithms [54]. The outcome of the algorithm is based on the predictions of the decision trees. The random forest consists of multiple individual decision trees [55]. Each of these trees operates as an ensemble. Although a random forest algorithm can cope with continuous values for regression and categorical values for classification, it provides better results for classification problems [55]. Therefore, we used the random forest algorithm in this study.

**Figure 8:**
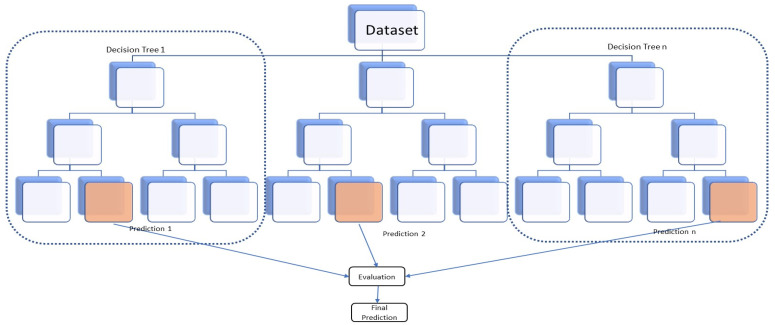
The random forest algorithm generates n number of decision trees, which takes subsets of the input dataset for training. The model’s final prediction will be the average of the outputs (1 to n) or the output with the highest number of votes in regression and classification, respectively.

Each tree is fed with the training dataset, with observations and features, to train themselves. Features are randomly selected during the splitting of the nodes. Every decision tree consists of decision nodes, leaf nodes, and a root node. Each decision tree in the random forest takes a subset of data to train itself and makes predictions accordingly (Figure 8). In classification, the class with the most votes represents the model’s final prediction. Conversely, the average of the predictions becomes the model’s final prediction.

One of the biggest problems associated with machine learning is overfitting. Since the random forest uses ensemble learning, it creates as many trees as possible. Each tree is trained using a subset of the whole dataset. This practice reduces overfitting and increases accuracy. The algorithm automatically handles missing values and outliers in the dataset. Hence, the algorithm is less impacted by noise in the input dataset. Normalisation or standardisation of the dataset is not required as, unlike most other algorithms, the random forest method does not use distance calculations; instead, it uses a rule-based approach. The random forest algorithm also explains the importance of input features.

Despite the advantages associated with this approach, the random forest method requires more training time than other algorithms because it creates a lot of decision trees. Hence, this process requires more computational power and resources.

### 3.1. Viticulture to Predict Yield Model

**Figure 9:**
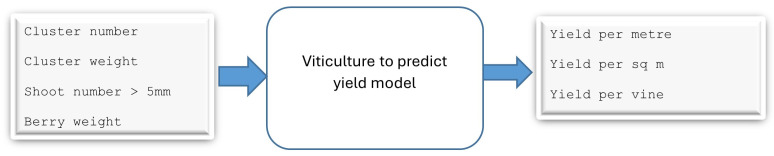
Model 1: predictive model for forecasting yield from viticulture data.

As shown in Figure 9, the first model takes four input features and gives three output parameters. Cluster weight and berry weight were measured in grams and the outputs were given in kilograms. The reason for selecting these input features is that cluster number, cluster weight, shoot number, and berry weight can be measured in vineyards. The outputs are the yield in different measurements. The synthetic data (6000 samples) were initially split into three datasets for training, validation, and testing, at a ratio of 6:2:2.We used deep learning with multilayer perceptron modelling techniques and the random forest algorithm to develop the model. We used the R2 score to measure the model’s accuracy viticulture to predict the juice parameters model.

**Figure 10:**
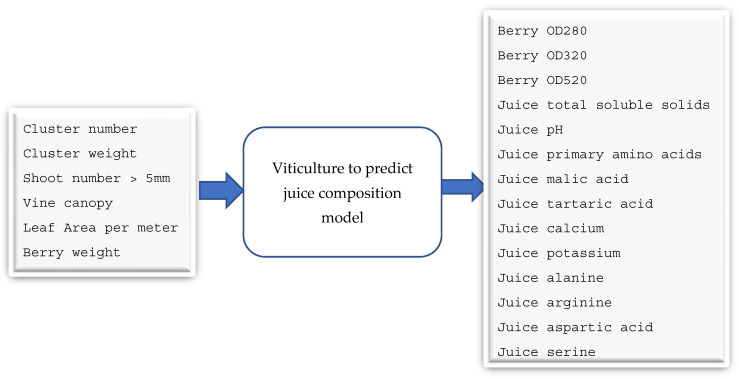
Model 2: predictive model for forecasting selected juice parameters from viticulture data.

As shown in Figure 10, the second model takes six input features and gives 14 output parameters. Again, these inputs are measurable, whereas the outputs are based on model 2. Cluster weight and berry weight were measured in grams and vine canopy was the percentage of canopy in the whole vine. Leaf area was measured in square centimetres. Optical density values were measured in absorbance units and total soluble solids were in °Brix. Primary amino acids, malic acid, and tartaric acid were measured in grams per litre and calcium and potassium were measured in milligrams per litre. Alanine, arginine, aspartic acid, and serine was measured in micromoles per litre. The synthetic data (6000 samples) were initially split into three datasets for training, validation, and testing, at a ratio of 6:2:2. We used deep learning with multilayer perceptron modelling techniques and the random forest algorithm to develop the model. We used the R2 score to measure the accuracy of the model.

### 3.2. Juice-Parameters-to-Wine-Parameters Model

**Figure 11:**
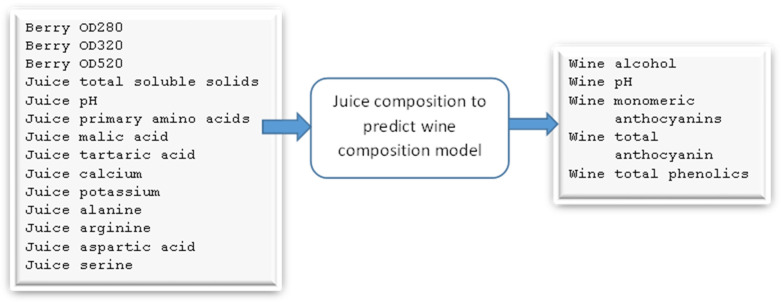
Model 3: predictive model for forecasting selected wine parameters from juice parameters.

As shown in Figure 11, the third model takes 14 input features and gives five output parameters. Optical density values were measured in absorbance units and total soluble solids were in °Brix. Primary amino acids, malic acid, and tartaric acid were in grams per litre, and calcium and potassium were measured in milligrams per litre. Alanine, arginine, aspartic acid, and serine were measured in micromoles per litre. Wine alcohol was measured as a percentage of alcohol volume per wine volume and anthocyanin values were measured in milligrams per litre. The synthetic data (6000 samples) were initially split into three datasets for training, validation, and testing, at a ratio of 6:2:2. We used deep learning with a multilayer perceptron modelling technique and a random forest algorithm to develop the model. We used the R2 score to measure the accuracy of the model.

### 3.3. Wine Parameters for Predicting the Quality of the Wine Product Model

**Figure 12:**
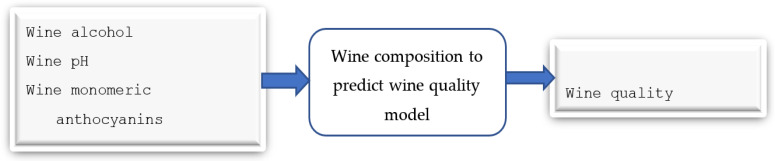
Model 4: predictive model for forecasting wine quality from wine parameters.

**Figure 13:**
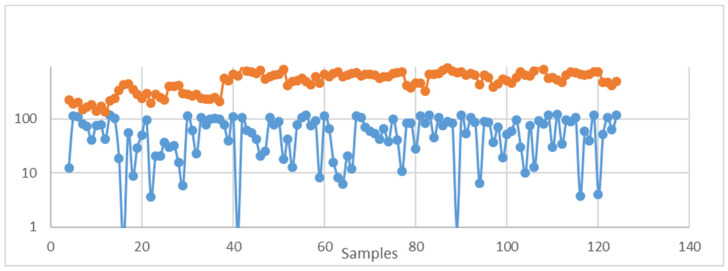
Anthocyanin trends based on statistical properties. The *y*-axis is the level of anthocyanin and the *x*-axis is the sample number.

**Figure 14:**
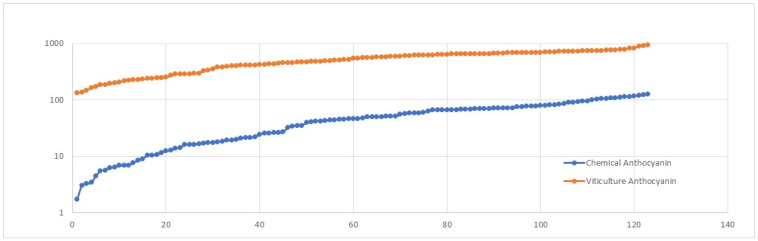
Trends based on statistical measures and the probability factor for each range of anthocyanin levels. *y*-axis: the level of anthocyanin; *x*-axis: the sample number.

The major issue with designing this model (Figure 12) was the absence of quality information in the original dataset. To overcome this issue, we analysed trends related to anthocyanin content: we used the original viticulture dataset and another dataset comprising the chemical composition and quality indices of a set of Pinot Noir wine samples. The anthocyanin content in wine comes from the fermentation and maceration of grapes. We retrieved the anthocyanin content of 18 samples of wines with quality values from previous research [10]. We synthesised 123 samples and analysed 18 samples based on basic statistical measures of mean and standard deviation. In addition, we categorised the range of anthocyanin values into bins (0–19.99, 20–39.99, 40–59.99, …). We considered the count of samples that lie in each bin when random samples were generated. We also included the probability count for each range. After we had synthesised the data, we plotted the trend for the anthocyanin values for both datasets (Figure 13 and Figure 14).

**Figure 15:**
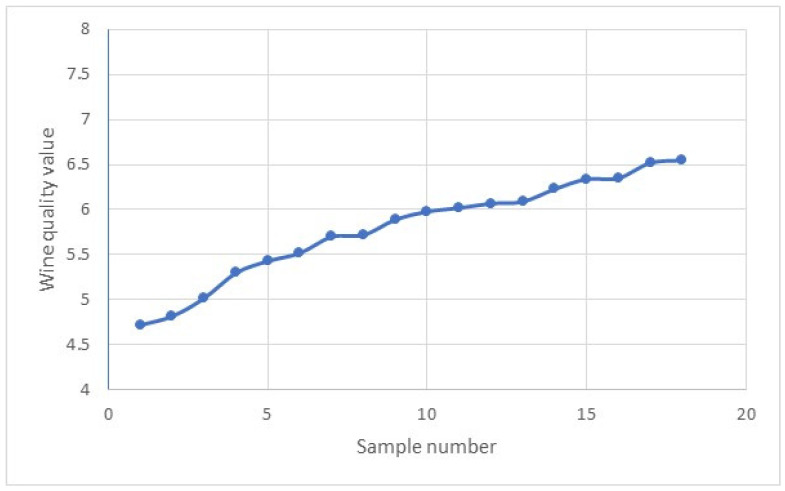
Wine quality trends for the 18 samples.

**Figure 16:**
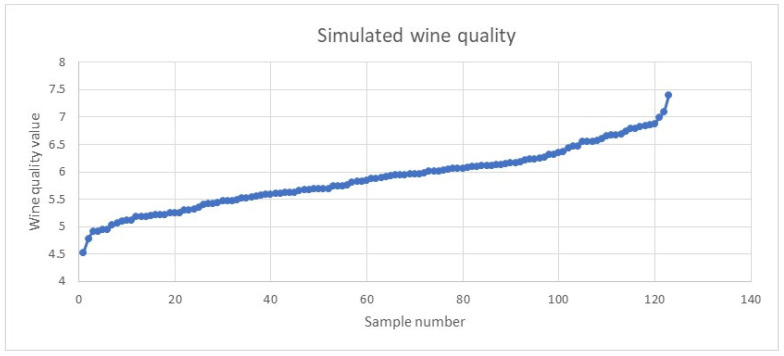
Wine quality trends in 123 synthesised samples.

Next, we generated the wine quality index for the 123 samples using lognormal distribution (simulated samples were within mean ± 1SD) based on the quality indices of 18 samples. Figure 15 and Figure 16 provide visual illustrations of the wine quality trends for the 18 samples and 123 synthesised samples.

The generated wine quality values were used as the quality indices for the 123 samples in the original dataset.

Wine alcohol was measured as a percentage of alcohol volume per wine volume and anthocyanin values were measured in milligrams per litre. The synthetic data (the 6000 samples) were initially split into three datasets for training, validation, and testing, at a ratio of 6:2:2. We used deep learning with a multilayer perceptron modelling technique and a random forest algorithm to develop the model. We used the R2 score to measure the model’s accuracy.

## 4. Discussion of the Results

We evaluated the deep learning models: those with different numbers of hidden layers (1, 2, and 3) and those with a different number of nodes of hidden layers (5, 10, 15, 20, and 25). We found that the R2 score does not change significantly, even where there are a significant number of hidden layers and multiple nodes. We also evaluated the deep learning model using different optimisation algorithms (adam, nadam, and SGD) and different activation functions for each of the layer nodes (tanh, ReLU, and ELU). We discovered that we could not significantly improve the model’s accuracy without including them.

**Figure 17:**
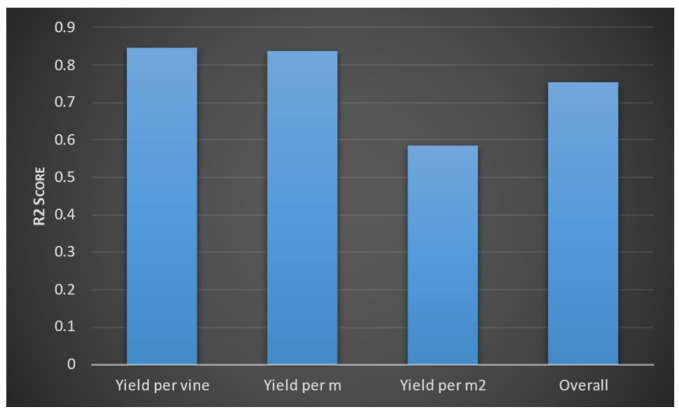
R2 values for the three outputs (yield per vine, yield per metre, and yield per square metre measured in kilograms) and the overall R2 score for the model’s accuracy.

**Figure 18:**
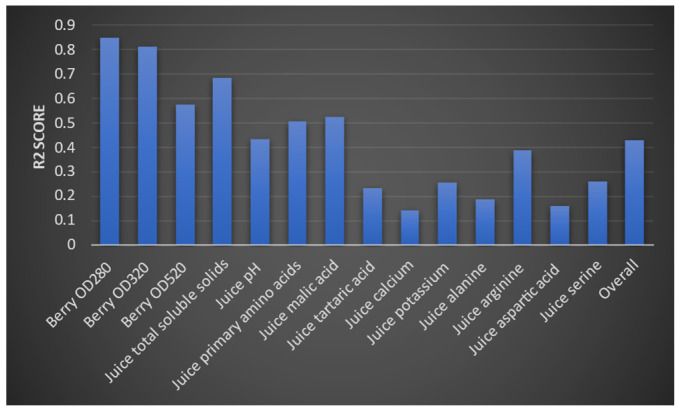
R2 values for the 14 outputs and the overall R2 score for the model’s accuracy.

**Figure 19:**
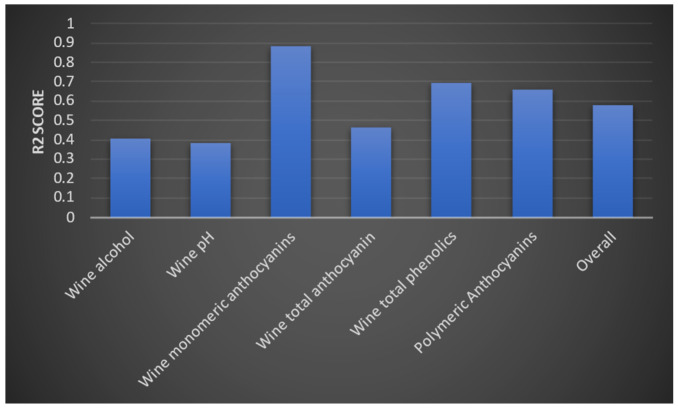
R2 values for the six outputs and the overall R2 score for the model’s accuracy.

We then evaluated the deep learning model and the random forest algorithm. We were only able to improve the accuracy of the models by a small value. We then evaluated each model using a test dataset. We measured accuracy using an R2 score. Model 1 obtained the following R2 values for the three outputs (Figure 17). According to the interpretation of R2 values, yield per wine and yield per metre have greater accuracy than yield per square metre. The model two evaluation results are provided in Figure 18. Accordingly, optical density values (at 280 and 320 mm wavelengths) and total soluble solids were predicted to have higher accuracy, whereas the berry optical density (at wavelength 520 mm), malic acid level, primary amino acid level, and pH values of grape juice were predicted with moderate accuracy. These were similar to the predictions from the third model, which are shown in Figure 19. Levels of monomeric anthocyanins, total phenolics, and polymeric anthocyanins were predicted with higher accuracy; others had a moderate level of accuracy. The fourth model had the highest R2 score: 0.999.

### Face Validation of the Models

**Figure 20:**
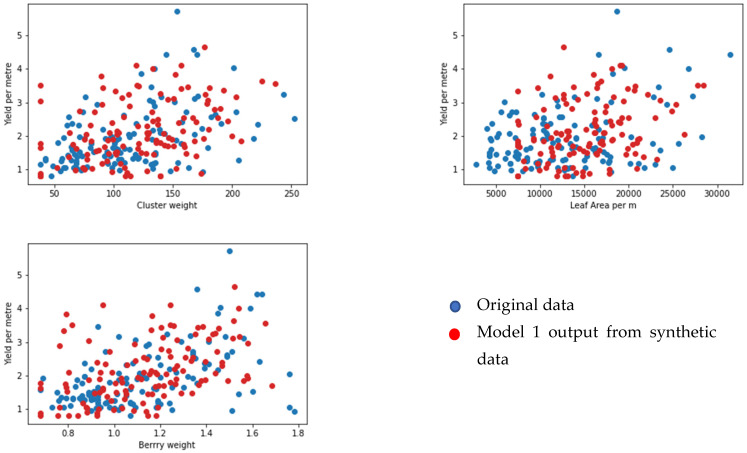
Plot one output from model 1 (yield per metre in kilograms) against three inputs (cluster weight (g), leaf area per m (cm), and berry weight (g)).

**Figure 21:**
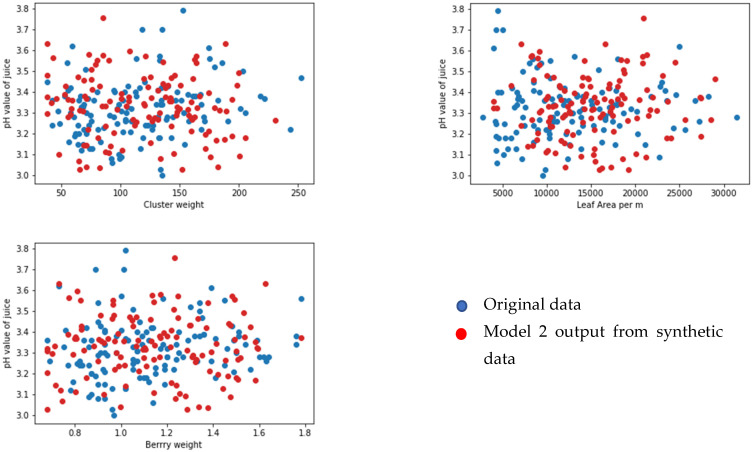
Plot one output from model 2 (pH value of juice) against three inputs (cluster weight (g), leaf area per m (cm), and berry weight (g)).

**Figure 22:**
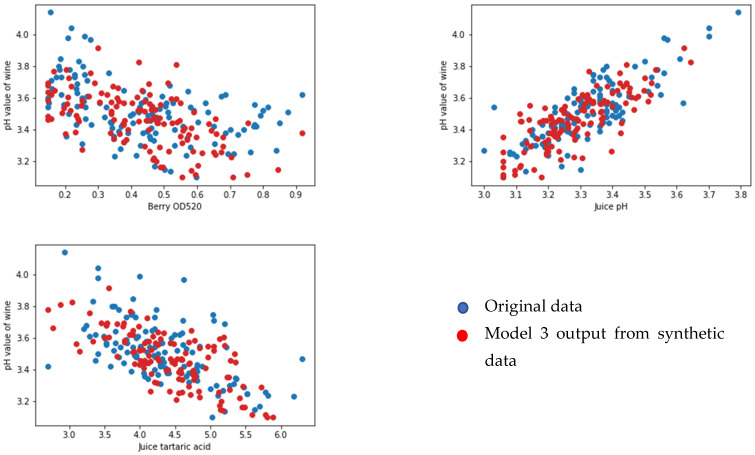
Plot one output from model 3 (pH value of berry juice) against three inputs (berry OD520 (AU), juice pH, and juice tartaric acid (g/L)).

**Figure 23:**
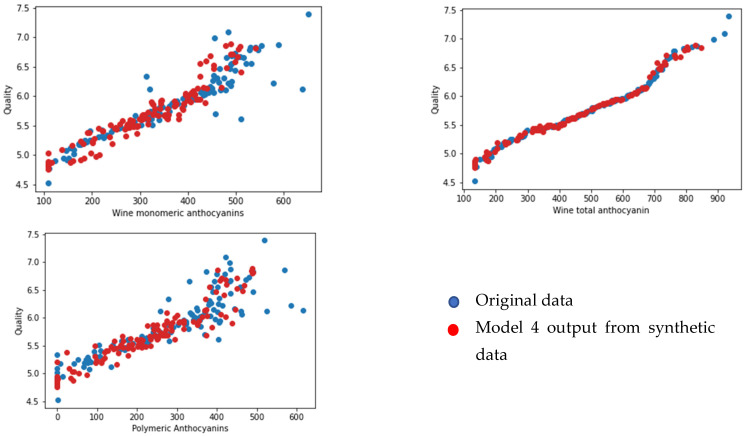
Plot one output from model 4 (wine quality) against three inputs (wine monomeric anthocyanin (mg/L), wine total anthocyanin (mg/L), and polymeric anthocyanin (mg/L)).

We selected 123 samples from the synthetic dataset. We simulated each model to predict the values for corresponding output parameters. We then compared the output data from the original dataset that were set aside for the face validation using random sampling. We selected one output from each model (yield per metre from model 1, pH value of grape juice for model 2, pH value of wine product for model 3, and quality of wine product from model 4). We compared the output from the models and the linked feature in the original dataset against three input features for each model (see Figure 20, Figure 21, Figure 22 and Figure 23 below). According to face validation, if the new simulation output data compares closely with the system output data, then the model can be considered “valid” [56]. According to the figures, the simulation results are consistent with the expected system behaviour. In this case, the model is said to have face validity.

## 5. Development of a Web Application for the End User

Winemakers are always seeking improvements in wine quality. For this reason, technologies to improve the quality and quantity (yield) of wine have been invented, with continual improvements in accuracy and efficiency being made every year. Parameters are incorporated into the prediction model we have developed: vintage, the number of shoots, the number of leaves, vine canopy, and berry weight. This model needs to be accessible to producers and users who are interested in wine quality prediction. With this prediction model, improvement in wine quality and yield, exploiting cloud services and frameworks in Python (the computer language), becomes possible. We developed the web application based on the Streamlit cloud server and used the Streamlit framework with the Python language. We considered the user’s experience and the interface [57]. This report describes the cloud service technology and the Streamlit framework.

### 5.1. Cloud Service Technology

**Figure 24:**
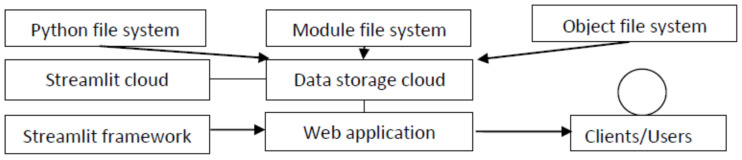
Components of cloud services.

Cloud computing contains information and application resources from the underlying infrastructure. This technology enables agility, collaboration, and easy accessibility to data, which optimises and enables efficient computing. However, security is a key concern, as users sometimes store their private data on the cloud [58]. As a result of these concerns, cloud services have improved the security of their systems [59]. There are three types of cloud storage: private cloud storage, public cloud storage, and hybrid cloud storage [60]. Private cloud storage was developed for a small number of users who need to customise and control their data. Public cloud storage is suitable for several users or those with unstructured data. Hybrid cloud storage is suitable for clients who need both types of storage. Clients can arrange their cloud service based on the number of users and the type of data that they need to store. The cloud storage system works as shown in Figure 24.

We used the public cloud service that offers data confidentiality, availability, and integrity. We processed all the data on a selected volume: approximately 1 GB, with files and folders included. We used Linux, an operating system provided by the Streamlit cloud server.

### 5.2. The Streamlit Framework

Streamlit is an open-source framework that people can use to create a custom web app. It also supports the development of machine learning and data science. According to the user experience and user interface, Streamlit provides several ways to represent results as outputs; for example, adding normal text, content, and pivot charts. For input data, Streamlit provides straightforward source code to create interactive features such as checkboxes, select boxes, and sidebars. An important aspect of Streamlit is that developers who do not have front-end knowledge can build attractive user interfaces in no time. Furthermore, the Python library attached to Streamlit allows developers, or those who are into data science, to create and deploy their models into Streamlit.

**Figure 25:**
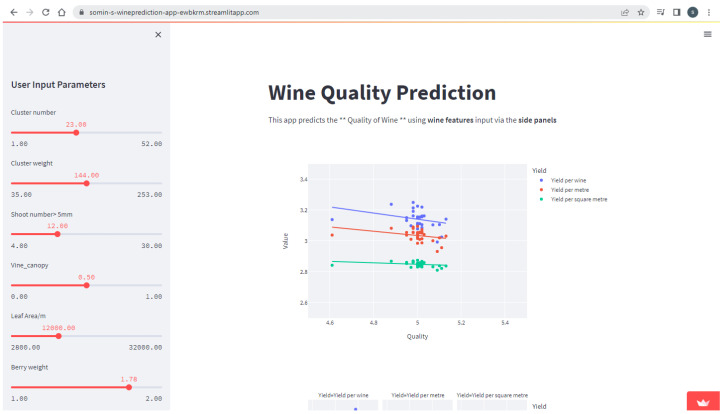
Web application for wine prediction.

We executed Streamlit in the local machine using Anaconda, a programme that brings together Python and R programming languages. There is a desktop graphical user interface for Anaconda called Anaconda Navigator, which can launch applications without using command-line commands. We uploaded our source code into the public GitHub repository and we connected the GitHub repository to the Streamlit account. As seen in Figure 25, we created web application features: an input feature (sidebar) and an output feature (graph and tables).

Cloud services and the Streamlit framework are useful tools for anyone wanting to develop web applications. Producers and clients who are interested in wine quality and yield prediction may use this web application to help them make informed decisions. As a result of specific predictions, producers can analyse predicted values based on input parameters such as yield, wine alcohol, wine pH, and phenolics. The app also predicts anthocyanin values, which have been discussed in terms of wine composition and enological practice [61]. Furthermore, the web application can predict wine quality using the models we developed.

**Figure 26:**
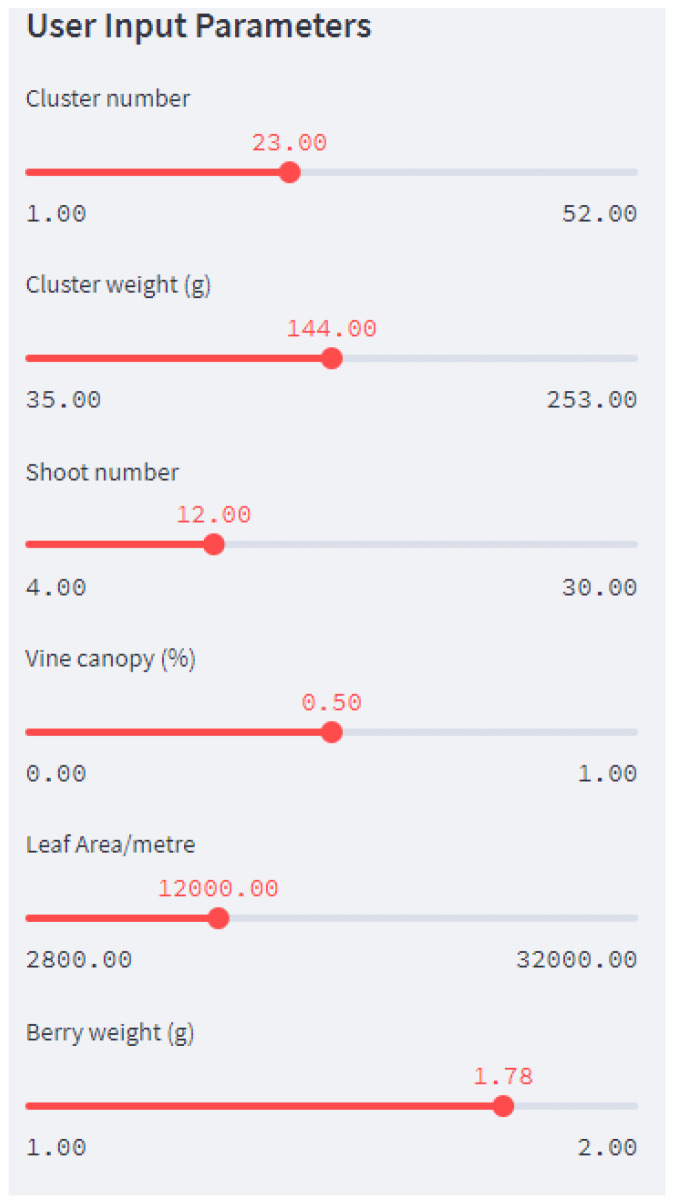
Input parameters for the web application.

**Figure 27:**
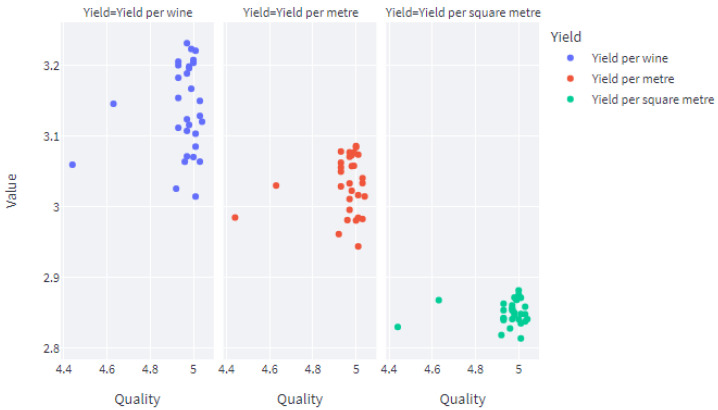
Output graphs from the web application.

Figure 26 shows how the user gives the expected average values for the input parameters. The application generates 20 sample sets of inputs from a normal (Gaussian) distribution based on the average values set by the user and the whole dataset is fed into the pipeline. It predicts yield per metre, yield per square metre, and yield per vine. It predicts the values for juice parameters and wine parameters. Finally, it predicts the expected quality of the wine product. The outputs of the pipeline, i.e., juice parameters and vine parameters, are visible to the user on request. In addition, Figure 27 shows how the predicted yield per meter, yield per vine and yield per square meter is plotted against the predicted quality.

**Figure 28:**
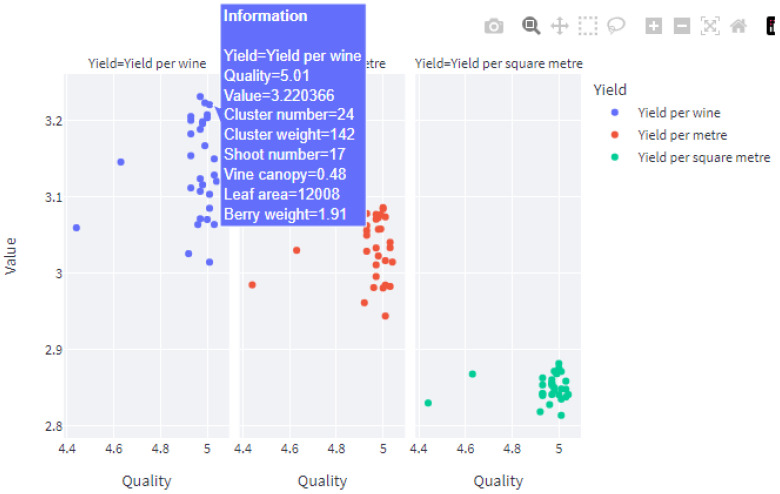
Viewing the values of input parameters for a certain point on the graph.

The web application provides insight into vineyard yield and the quality of the wine product based on the average values given for the viticulture parameters. The graphs show the different ways to balance the quality of the wine product and the yield of vineyards. From the generated datasets, if the user wants to know the values of the input parameters that satisfy the expected quality and yield values, the user can hover the mouse pointer on the selected point in the graph and view the corresponding values for the parameters, as in Figure 28. The web application can be found at https://wineprediction-dhe6sbowwzqzrwqpqbtscl.streamlit.app/ accessed on 22 September 2024.

## 6. Conclusions and Future Directions

Producing Pinot Noir presents challenges for viticulturists and winemakers. Machine learning techniques can be implemented in the wine industry to assess quality traits in the final product. With the proposed approach, we could design a pipeline that represents the wine-making process, beginning in the vineyard and ending with the final product. This proposed application would provide a powerful tool that winemakers could use to assess data from vineyards to determine grape juice characteristics and wine products from specific vineyards or regions. The vineyard owners could use the information provided by the tool to develop strategic solutions to balance their yield and the quality of their wine products. The web application mentioned here is available for any interested party to use at https://wineprediction-dhe6sbowwzqzrwqpqbtscl.streamlit.app/ accessed on 22 September 2024. We anticipate to further develop this tool as more data for Pinot Noir wines become available.

## Data Availability

The original contributions presented in the study are included in the article/Supplementary Material, further inquiries can be directed to the corresponding author.

## Supplementary Materials

The following supporting information can be downloaded at: https://www.mdpi.com/article/10.3390/foods13193091/s1, Figure S1: SHAP value summary plot for Yield per square meter; Figure S2: SHAP value summary plot for Yield per meter; Figure S3: SHAP value summary plot for Yield per vine; Figure S4: SHAP value summary plot for ODE280; Figure S5: SHAP value summary plot for ODE320; Figure S6: SHAP value summary plot for ODE520; Figure S7: SHAP value summary plot for alanine level in berry juice; Figure S8: SHAP value summary plot for ammonium level in berry juice; Figure S9: SHAP value summary plot for arginine level in berry juice; Figure S10: SHAP value summary plot for aspartic acid level in berry juice; Figure S11: SHAP value summary plot for Calcium level in berry juice; Figure S12: SHAP value summary plot for glutamic acid level in berry juice; Figure S13: SHAP value summary plot for magnesium level in berry juice; Figure S14: SHAP value summary plot for malic acid level in berry juice; Figure S15: SHAP value summary plot for pH level in berry juice; Figure S16: SHAP value summary plot for potassium level in berry juice; Figure S17: SHAP value summary plot for primary amino acids level in berry juice; Figure S18: SHAP value summary plot for serine level in berry juice; Figure S19: SHAP value summary plot for tartaric acid level in berry juice; Figure S20: SHAP value summary plot for threonine level in berry juice; Figure S21: SHAP value summary plot for titratable acidity level in berry juice; Figure S22: SHAP value summary plot for total soluble solids in berry juice; Figure S23: SHAP value summary plot for tyrosine level in berry juice; Figure S24: SHAP value summary plot for caffeic acid levels in wine; Figure S25: SHAP value summary plot for catechin levels in wine; Figure S26: SHAP value summary plot for epicatechin levels in wine; Figure S27: SHAP value summary plot for gallic acid levels in wine; Figure S28: SHAP value summary plot for Malvidin 3-glucoside levels in wine; Figure S29: SHAP value summary plot for Mark to wine ratio in wine; Figure S30: SHAP value summary plot for polymeric anthocyanin levels in wine; Figure S31: SHAP value summary plot for quercentin-G levels in wine; Figure S32: SHAP value summary plot for resveratrol levels in wine; Figure S33: SHAP value summary plot for trans caftaric acid levels in wine; Figure S34: SHAP value summary plot for Trans-coutaric acid levels in wine; Figure S35: SHAP value summary plot for alcohol levels in wine; Figure S36: SHAP value summary plot for color density levels in wine; Figure S37: SHAP value summary plot for hue of wine; Figure S38: SHAP value summary plot for methyl cellulose precipitable tannins levels in wine; Figure S39: SHAP value summary plot for monomeric anthocyanin levels in wine; Figure S40: SHAP value summary plot for pH value in wine; Figure S41: SHAP value summary plot for residual sugar levels in wine; Figure S42: SHAP value summary plot for titratable acidity levels in wine; Figure S43: SHAP value summary plot for total anthocyanin levels in wine; Figure S44: SHAP value summary plot for total phenolic levels in wine; Figure S45: SHAP value summary plot for the quality.
