# Supplementary material for: A Machine Learning Pipeline for Predicting Pinot Noir Wine Quality from Viticulture Data: Development and Implementation
Source: Foods. 2024 Sep 27;13(19):3091. doi: 10.3390/foods13193091 (PMC11476124; doi:10.3390/foods13193091)
Supplement: Supplementary file 1 [file foods-13-03091-s001.zip › foods-3191180-supplementary.pdf]

Supplementary Materials

S1: Shapely value summary plots

We have categorized the parameters into the four models and the SHAP value summary plots were drawn taking only the parameters in the relevant categories. For instance, yield parameters and parameters related to juice only depend on viticulture related data. Parameters related to wine composition depends on the parameters related to berry juice. The quality of the wine product is completely depend on the wine composition.

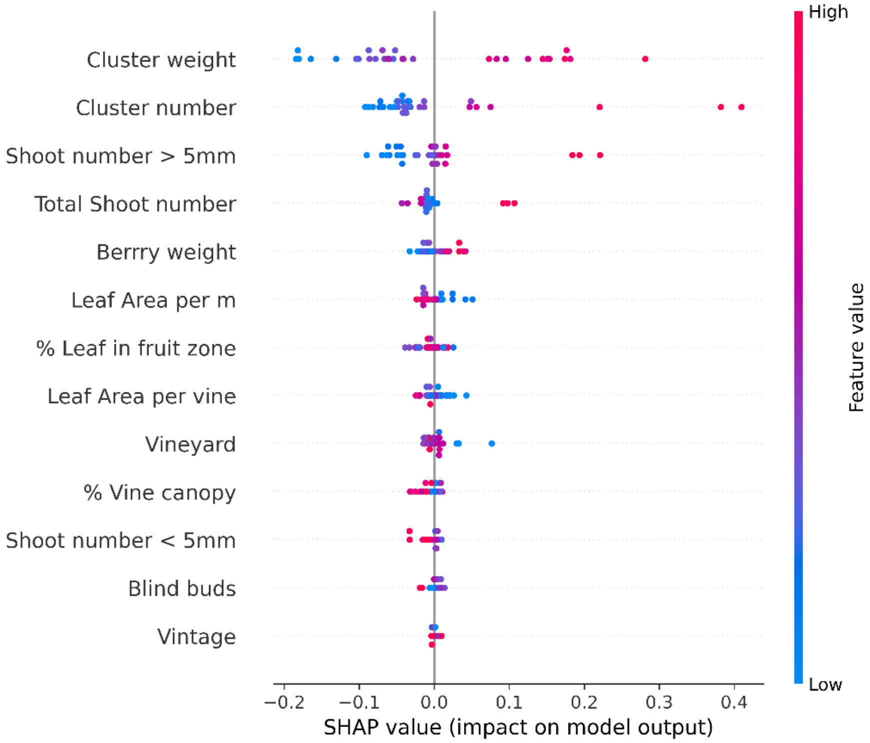

Figure S1: SHAP value summary plot for Yield per square meter

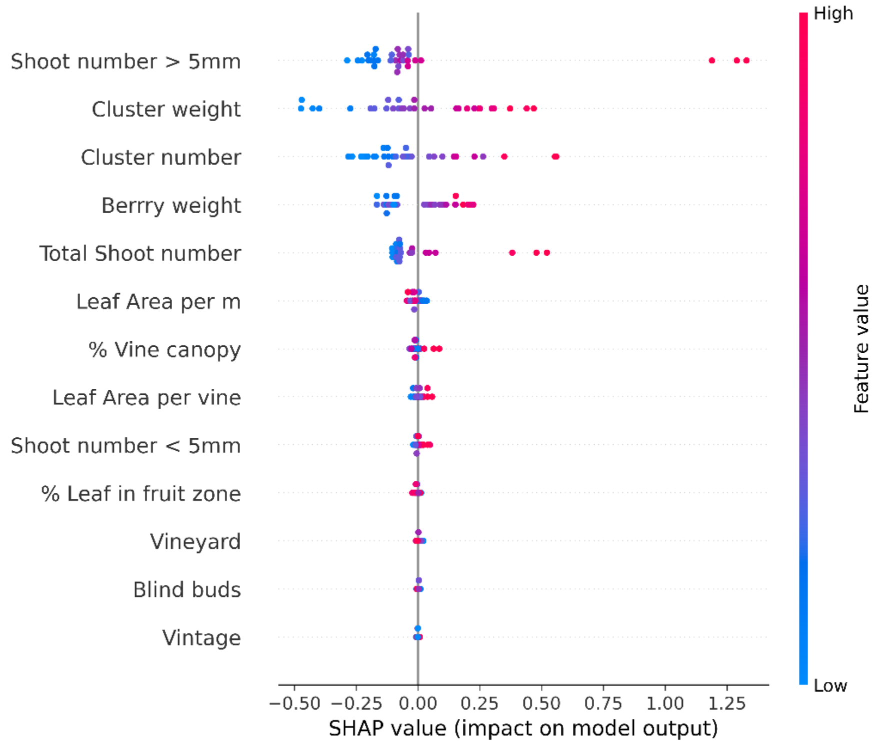

Figure S2: SHAP value summary plot for Yield per meter

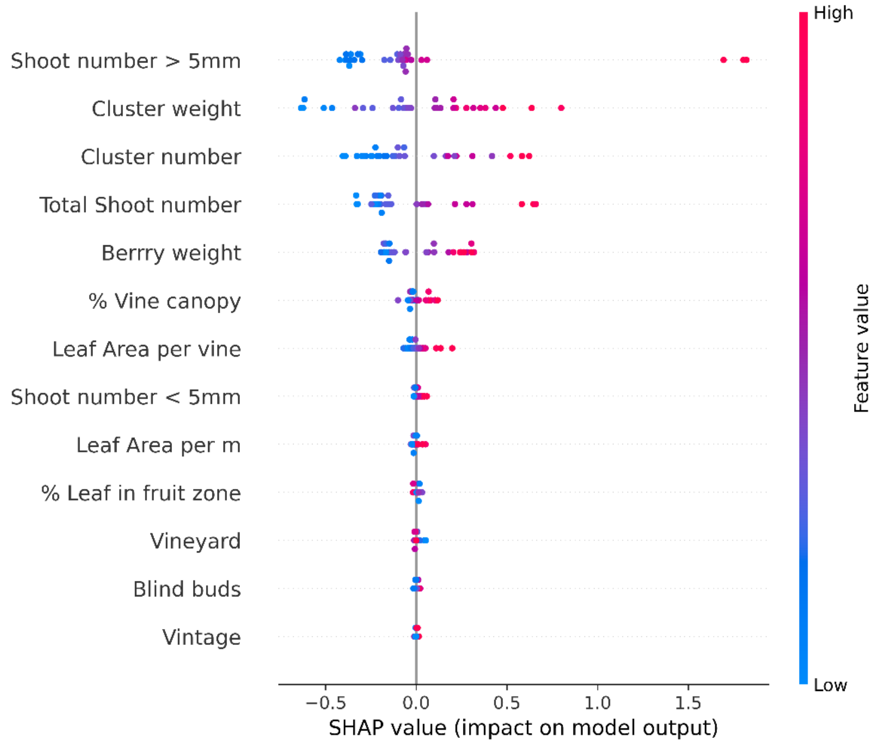

Figure S3: SHAP value summary plot for Yield per vine

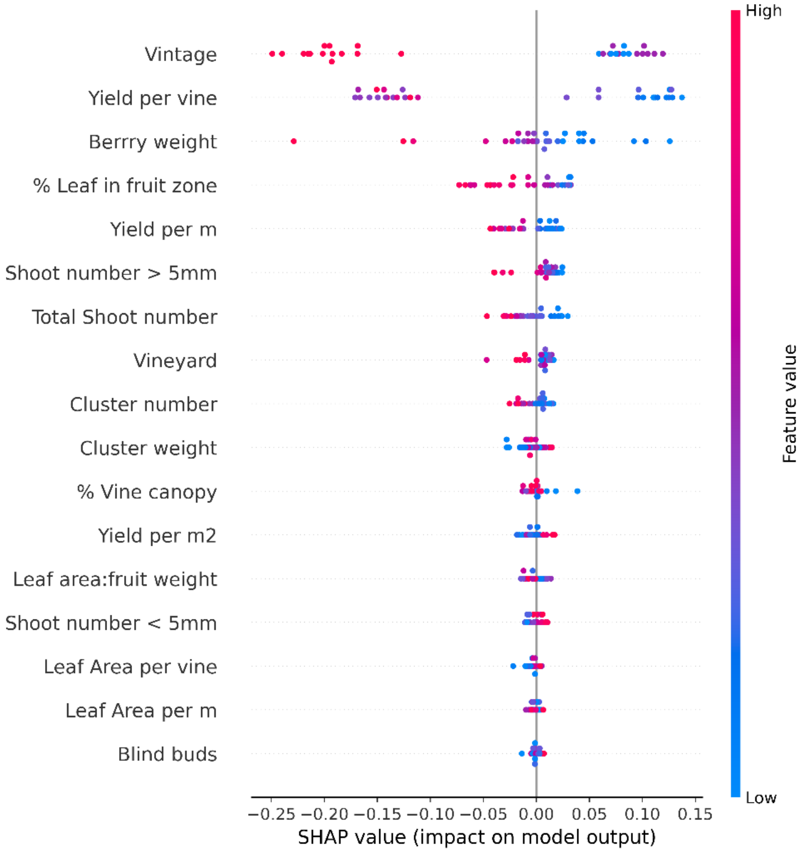

Figure S4:SHAP value summary plot for ODE280

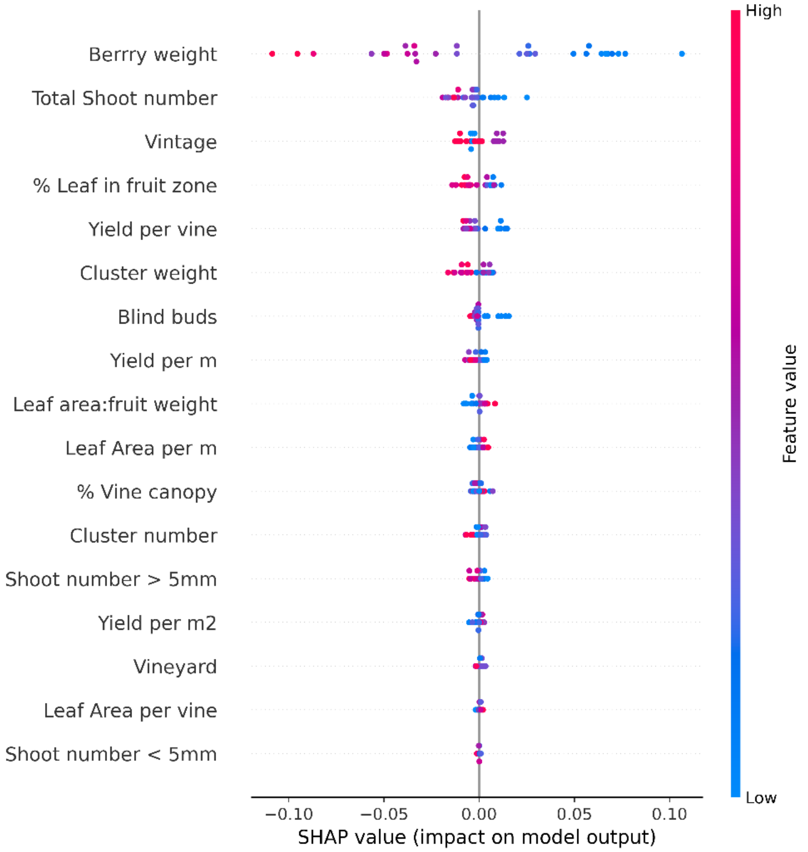

Figure S5: SHAP value summary plot for ODE320

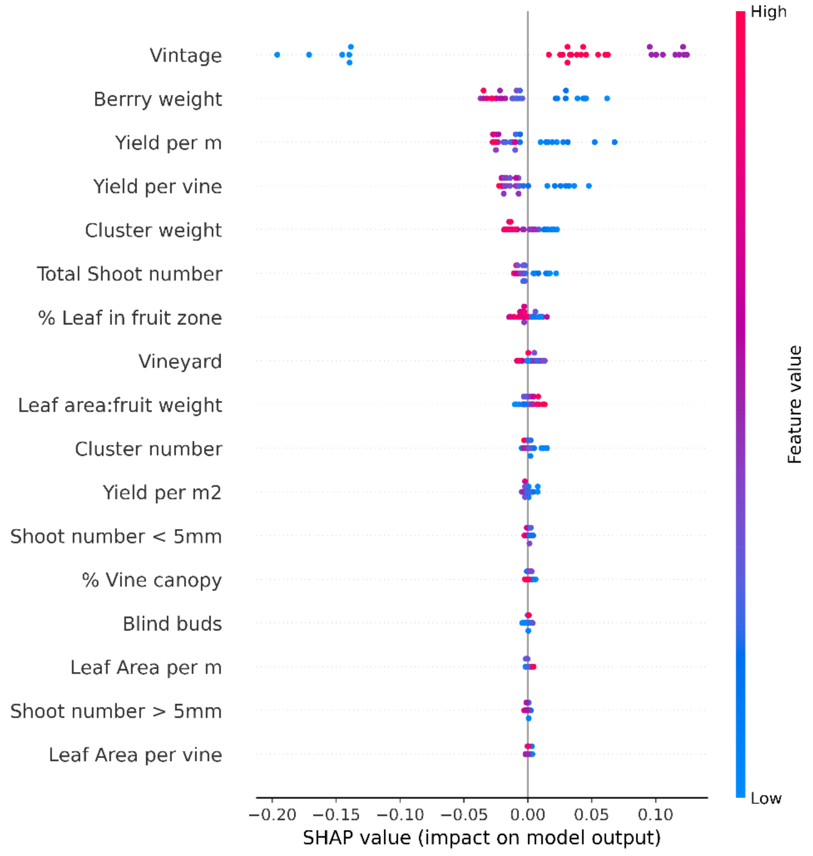

Figure S6: SHAP value summary plot for ODE520

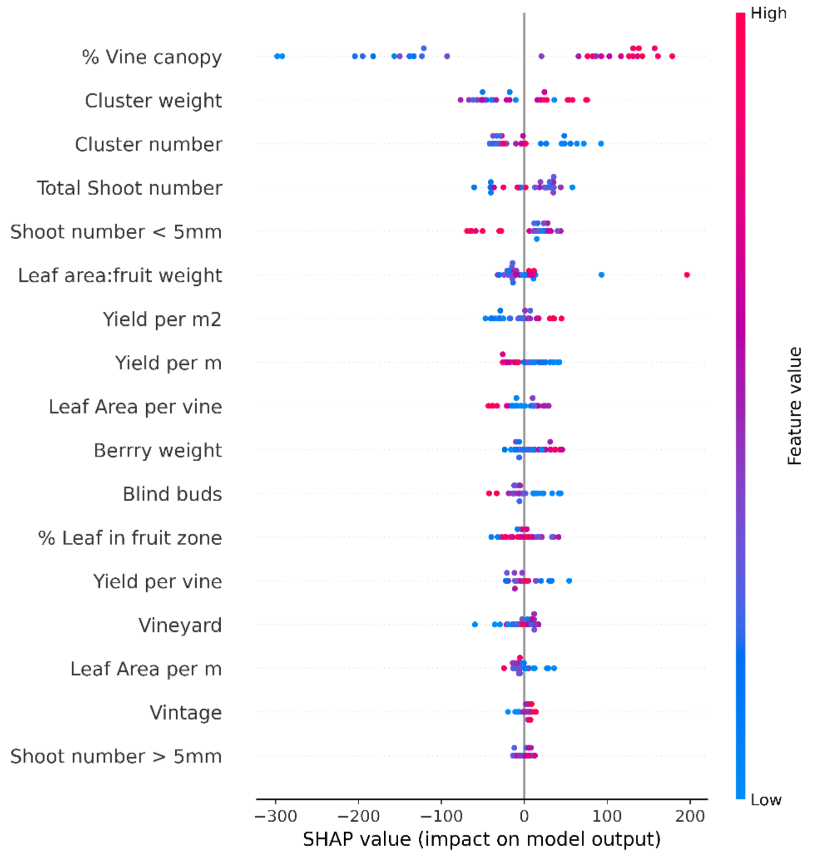

Figure S7: SHAP value summary plot for alanine level in berry juice

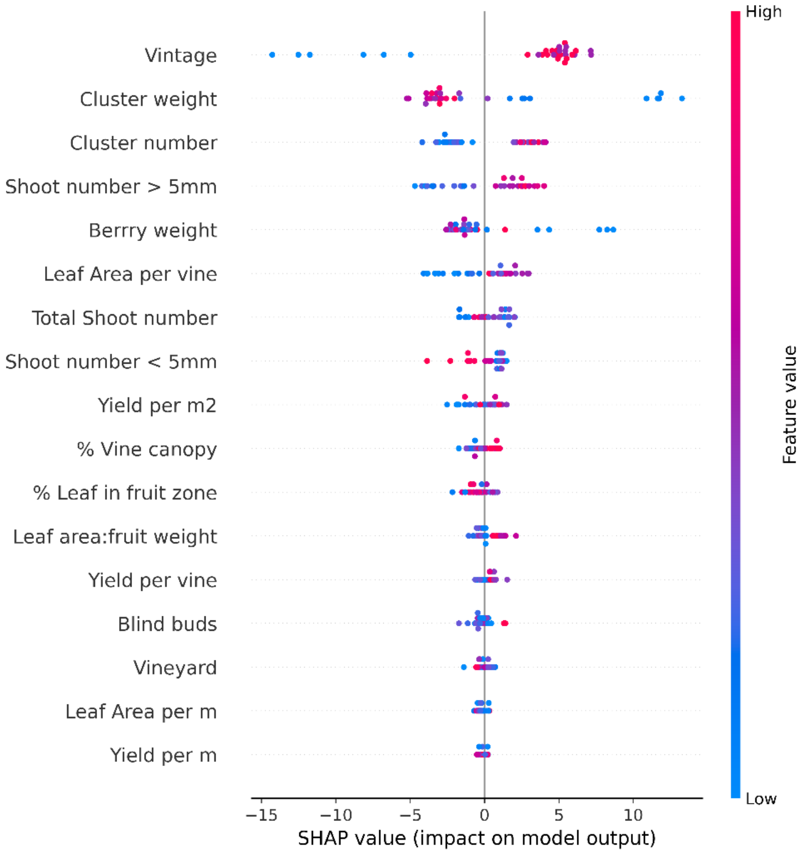

Figure S8: SHAP value summary plot for ammonium level in berry juice

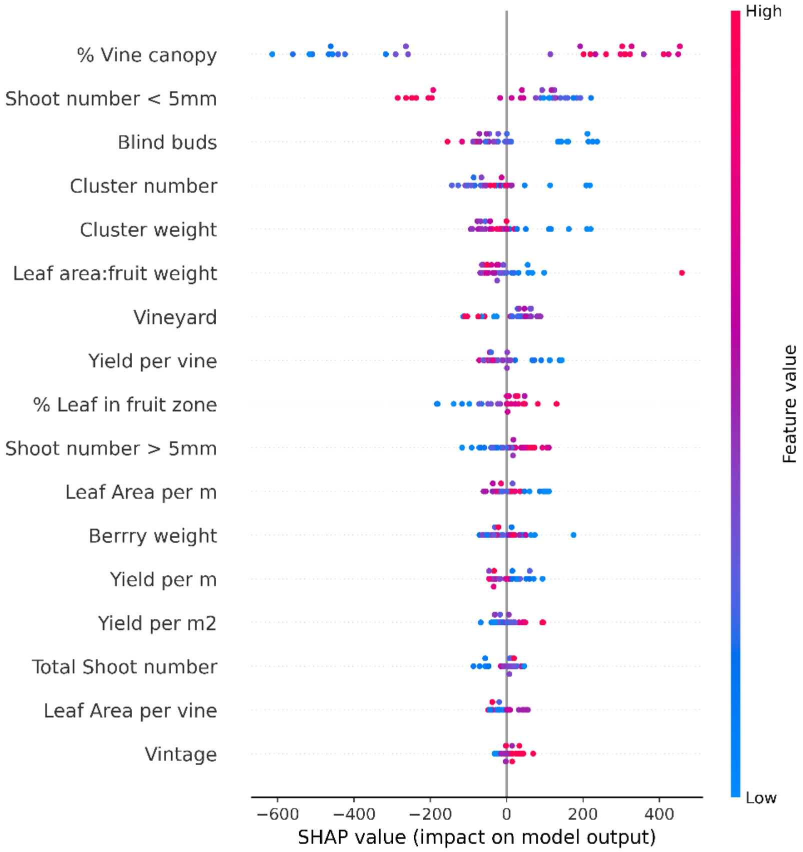

Figure S9: SHAP value summary plot for arginine level in berry juice

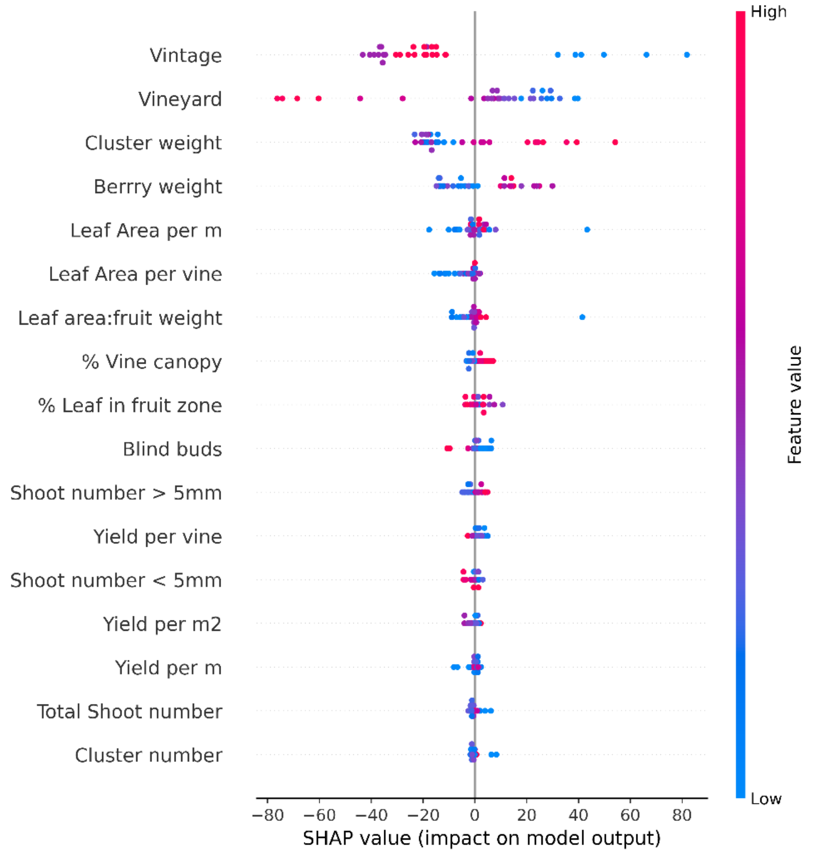

Figure S10: SHAP value summary plot for aspartic acid level in berry juice

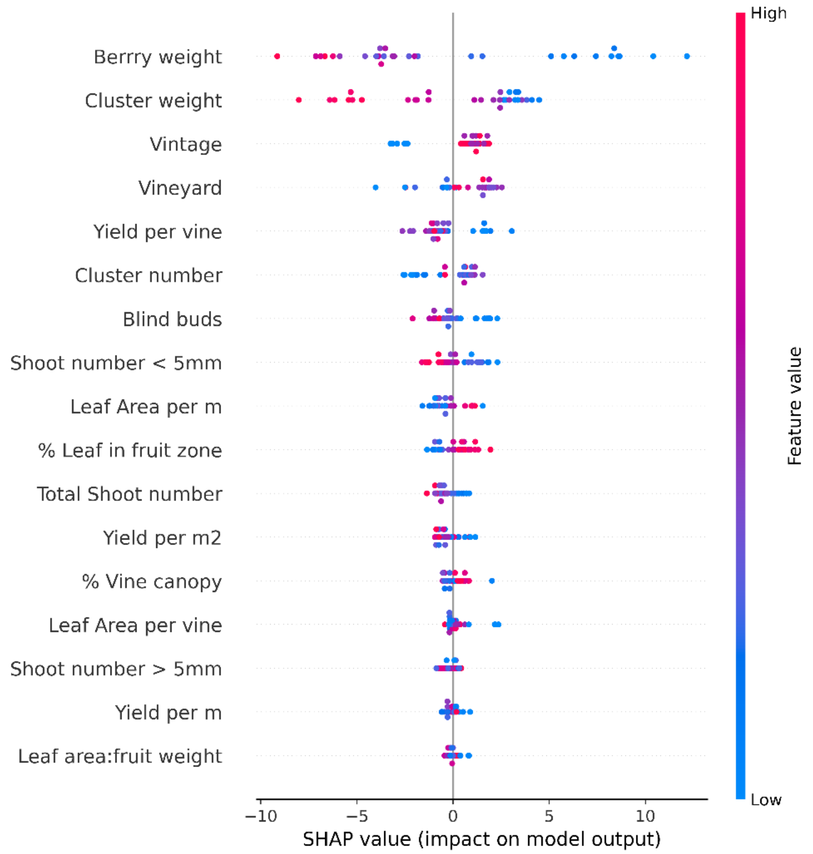

Figure S11: SHAP value summary plot for Calcium level in berry juice

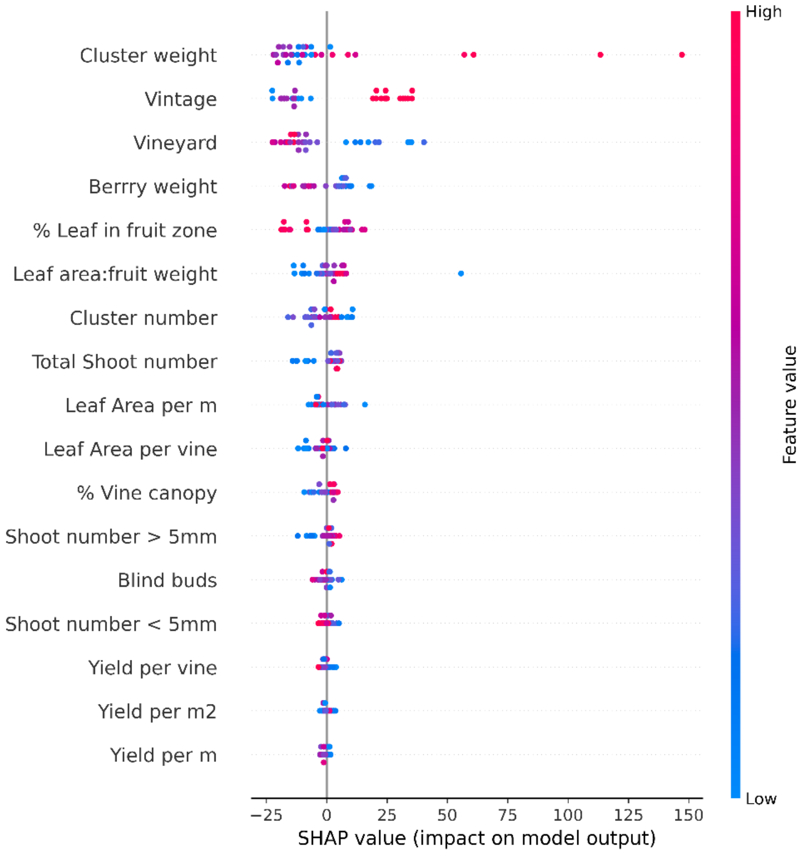

Figure S12: SHAP value summary plot for glutamic acid level in berry juice

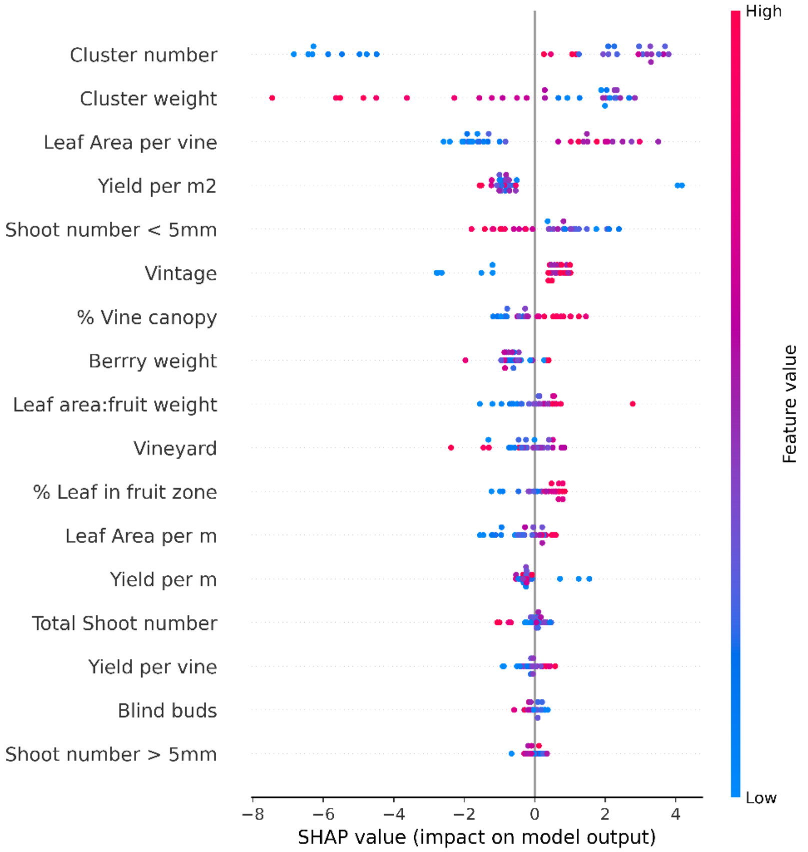

Figure S13: SHAP value summary plot for magnesium level in berry juice

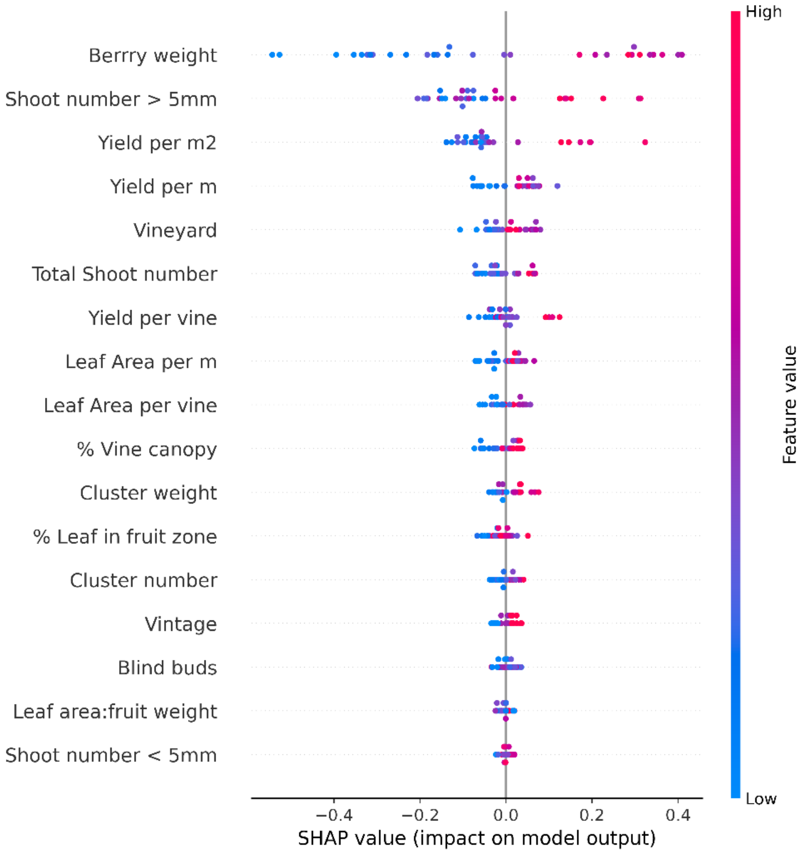

Figure S14: SHAP value summary plot for malic acid level in berry juice

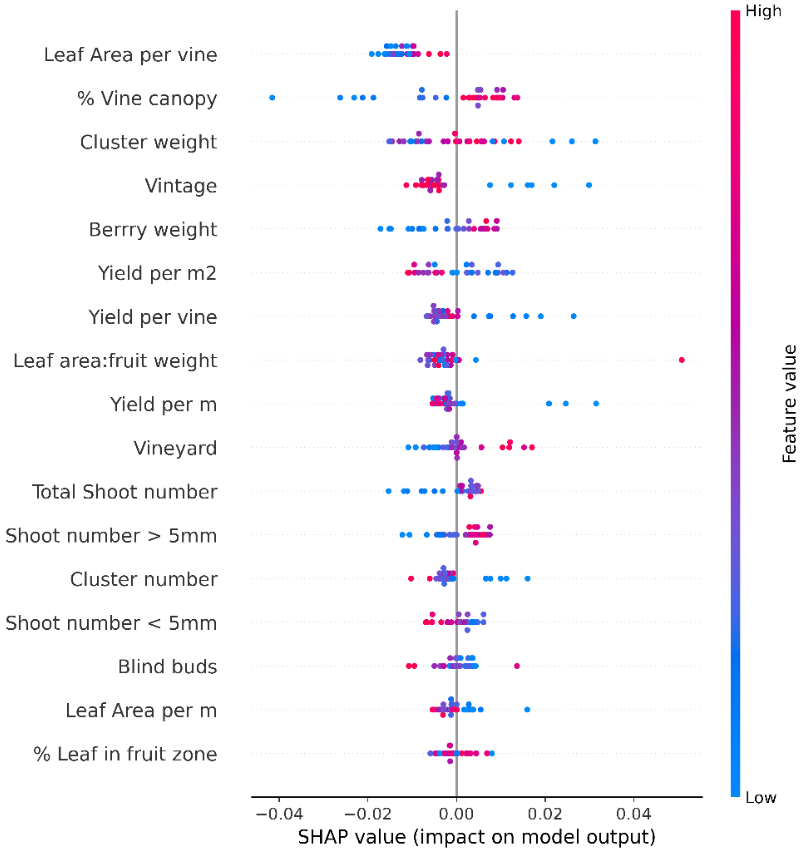

Figure S15: SHAP value summary plot for pH level in berry juice

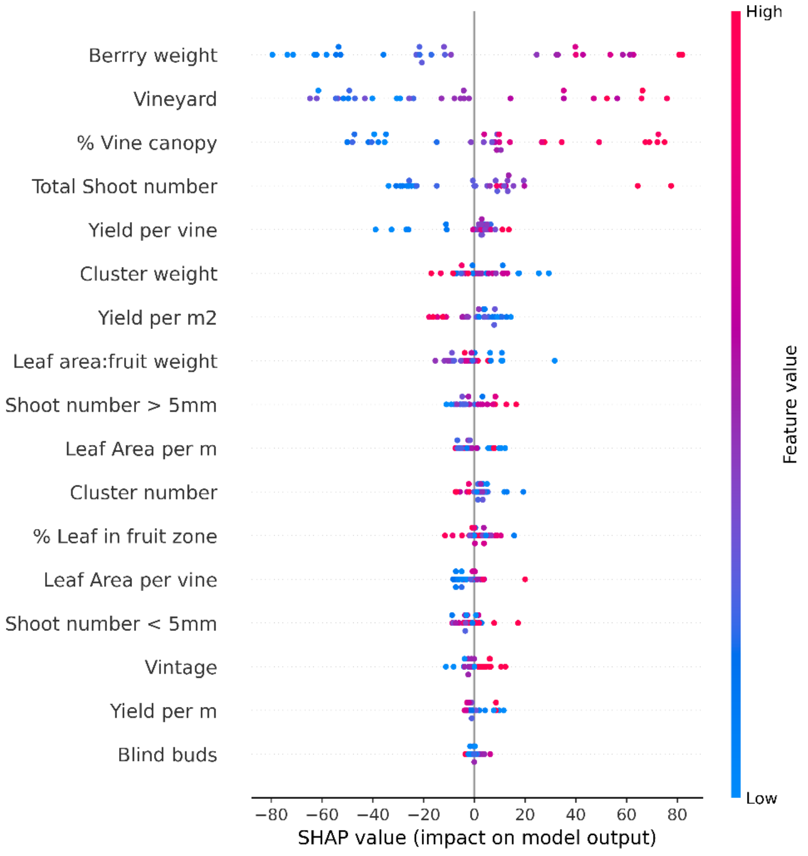

Figure S16: SHAP value summary plot for potassium level in berry juice

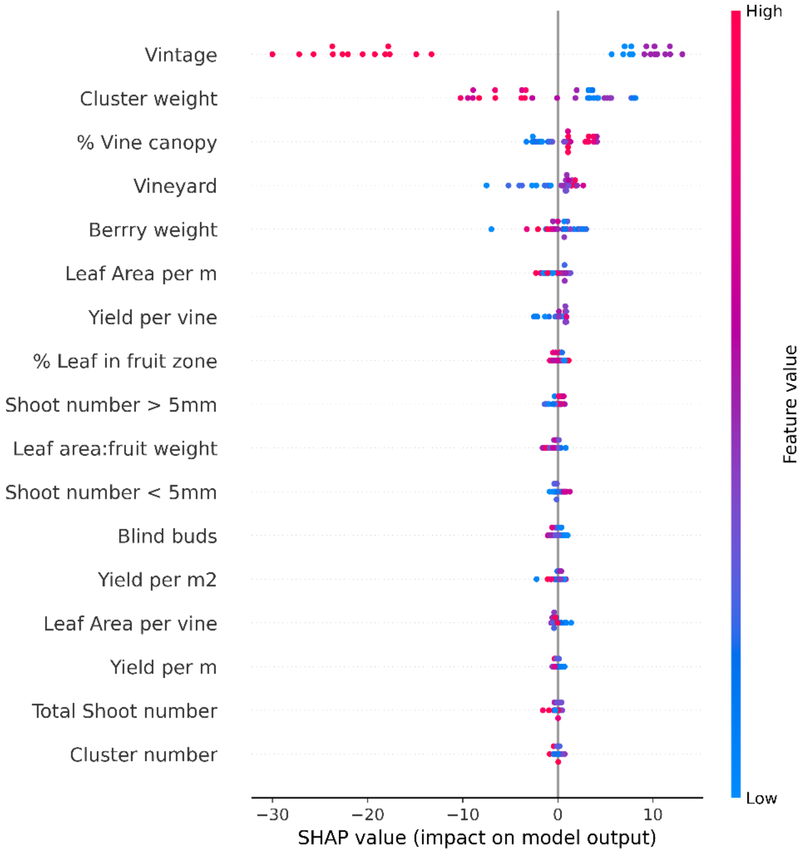

Figure S17: SHAP value summary plot for primary amino acids level in berry juice

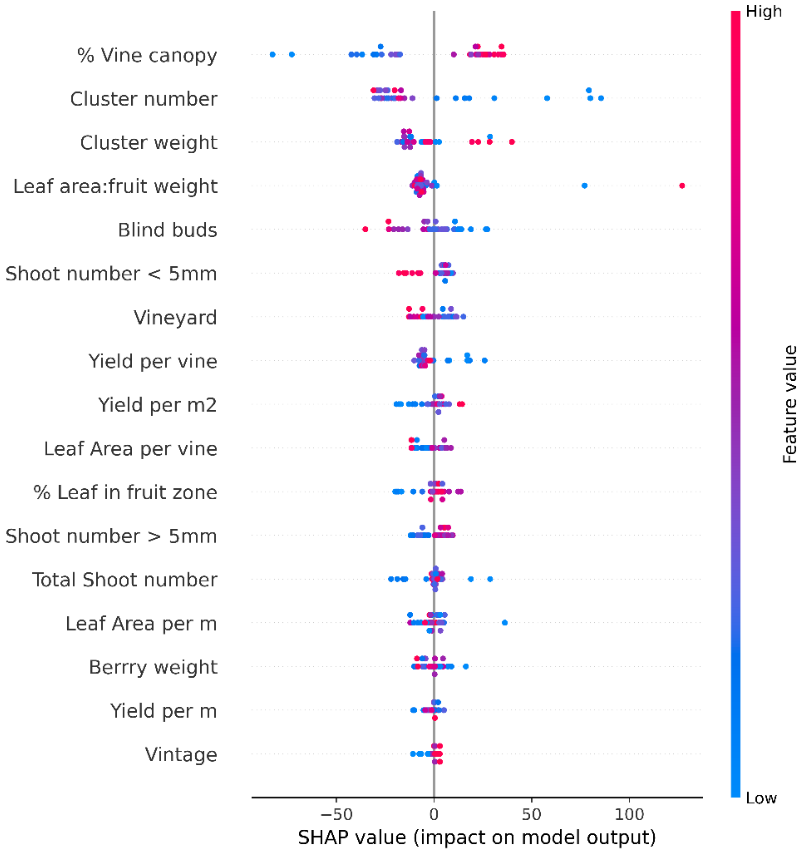

Figure S18: SHAP value summary plot for serine level in berry juice

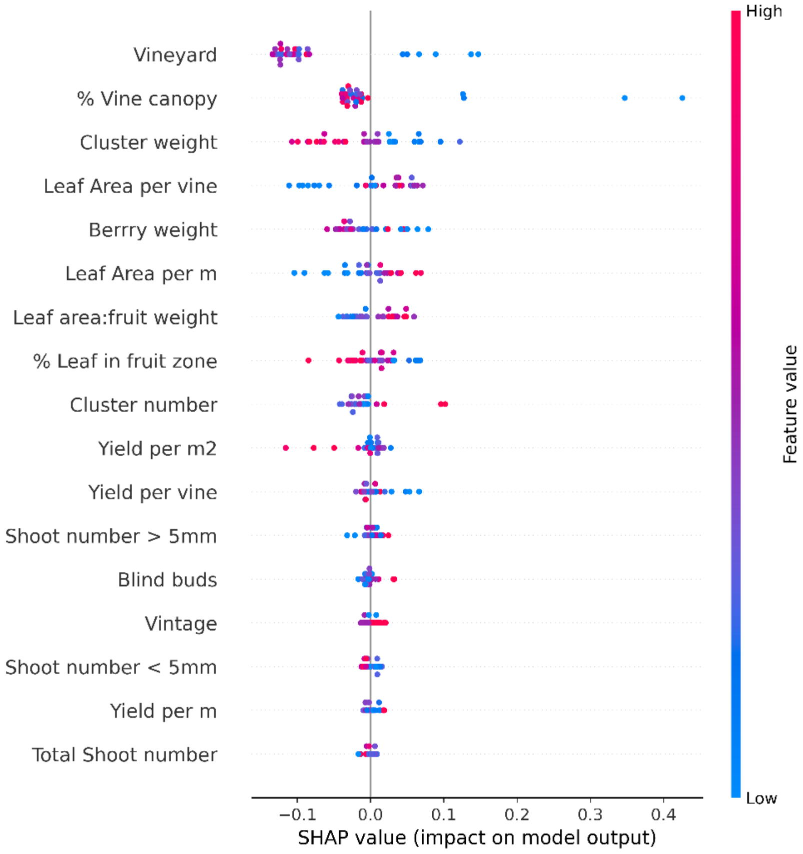

Figure S19: SHAP value summary plot for tartaric acid level in berry juice

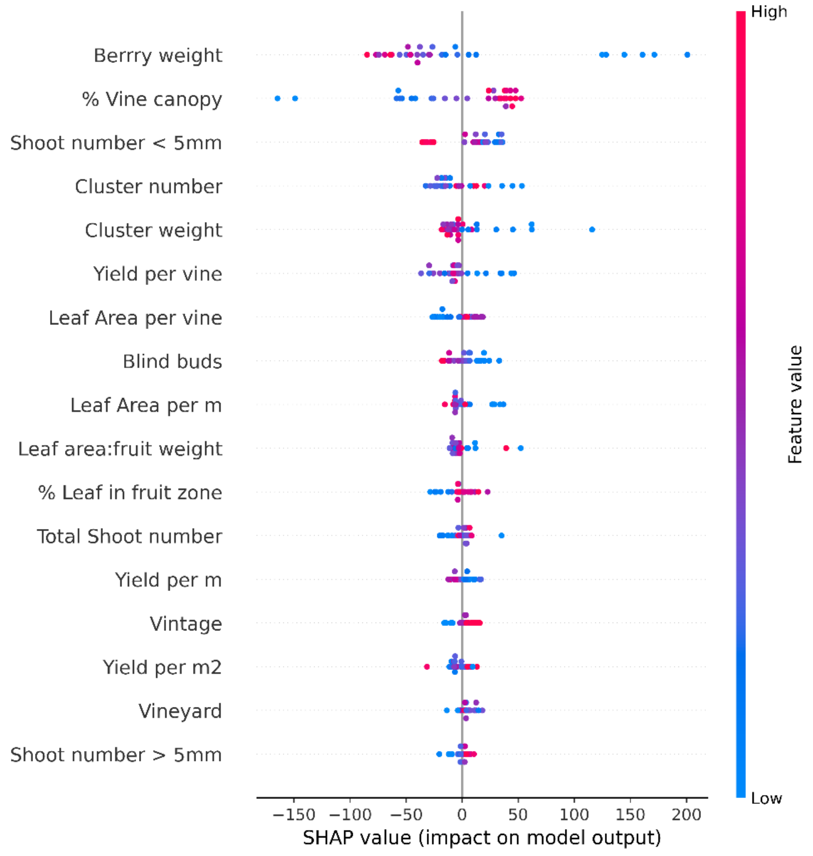

Figure S20: SHAP value summary plot for threonine level in berry juice

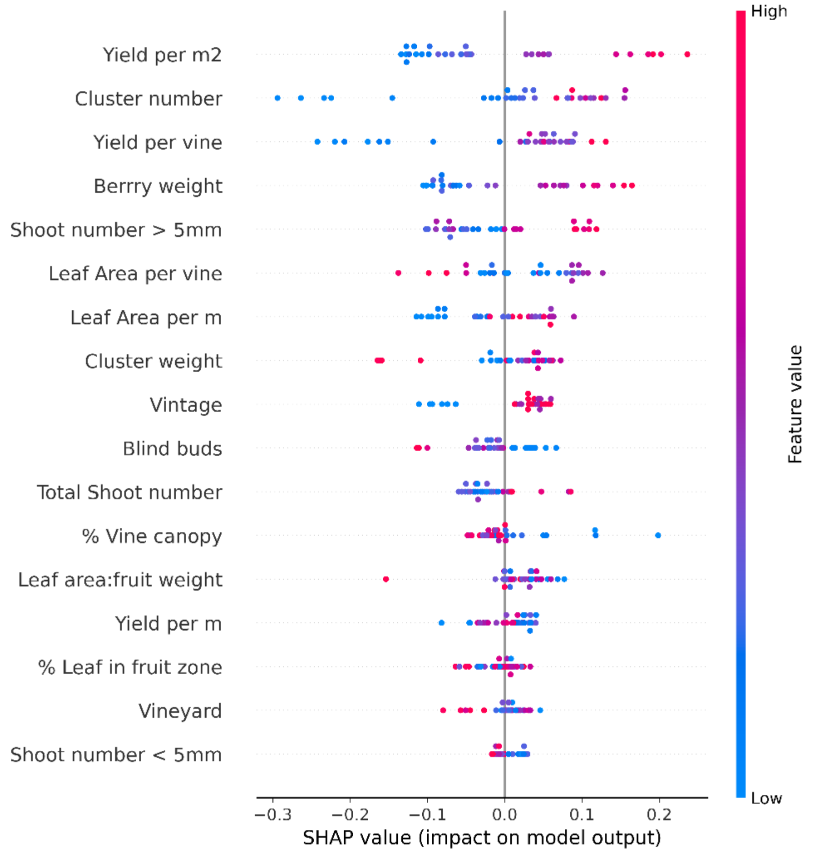

Figure S21: SHAP value summary plot for titratable acidity level in berry juice

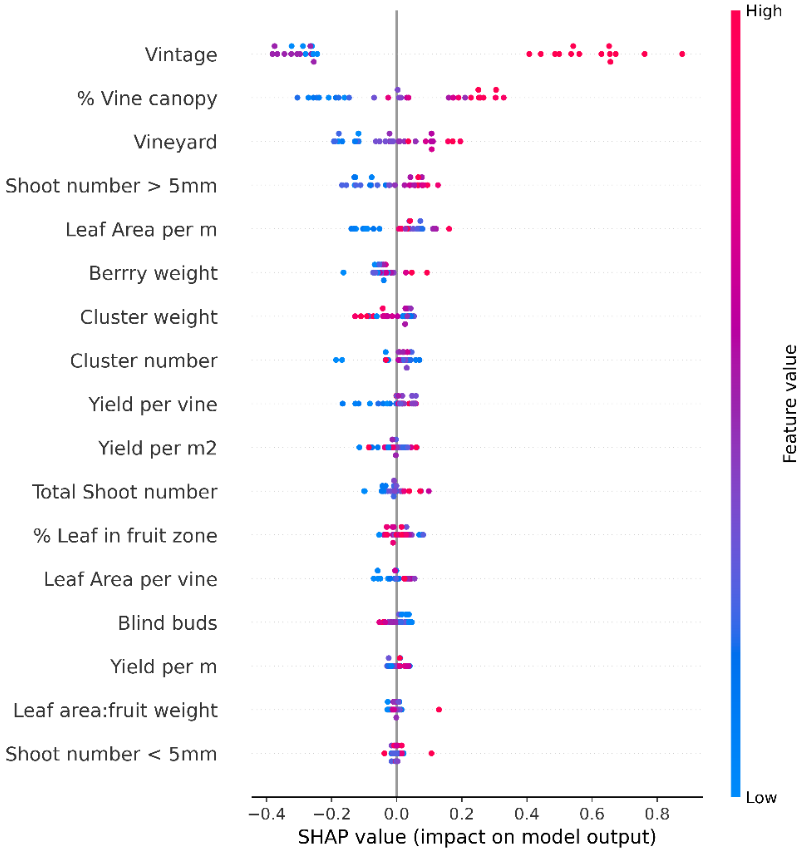

Figure S22: SHAP value summary plot for total soluble solids in berry juice

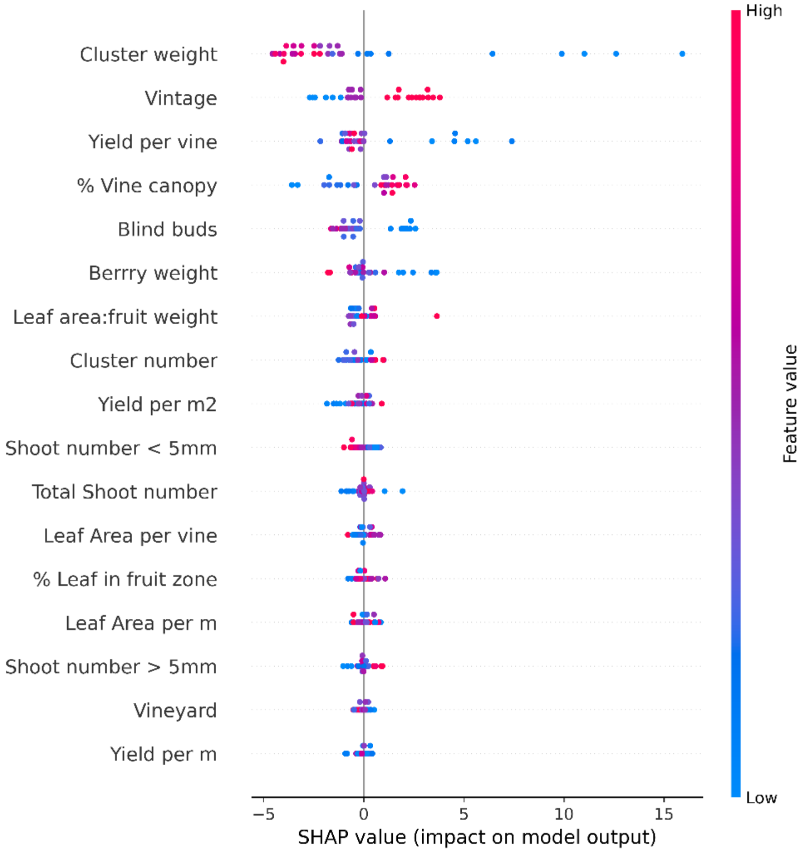

Figure S23: SHAP value summary plot for tyrosine level in berry juice

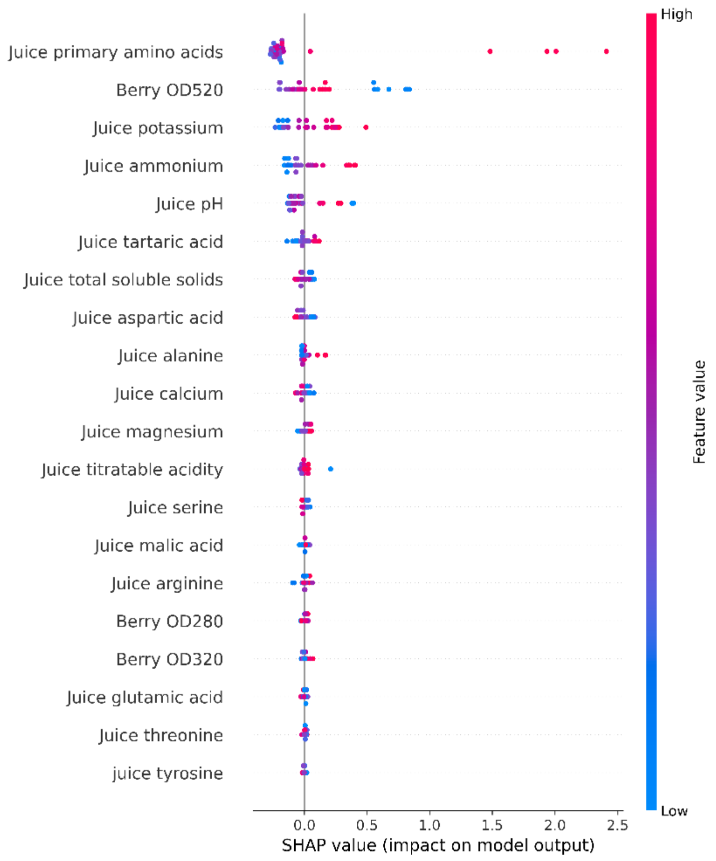

Figure S24: SHAP value summary plot for caffeic acid levels in wine

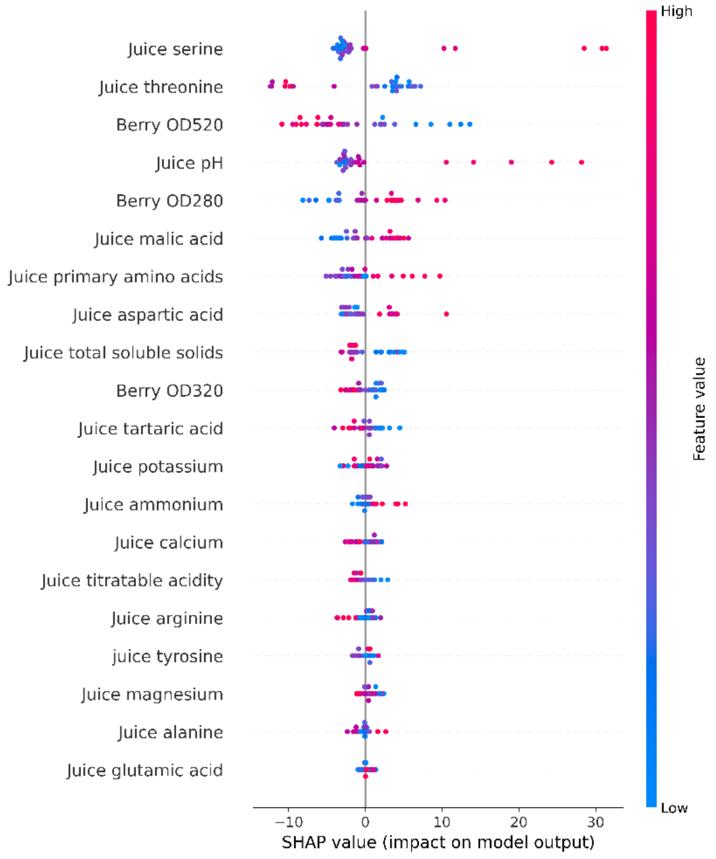

Figure S25: SHAP value summary plot for catechin levels in wine

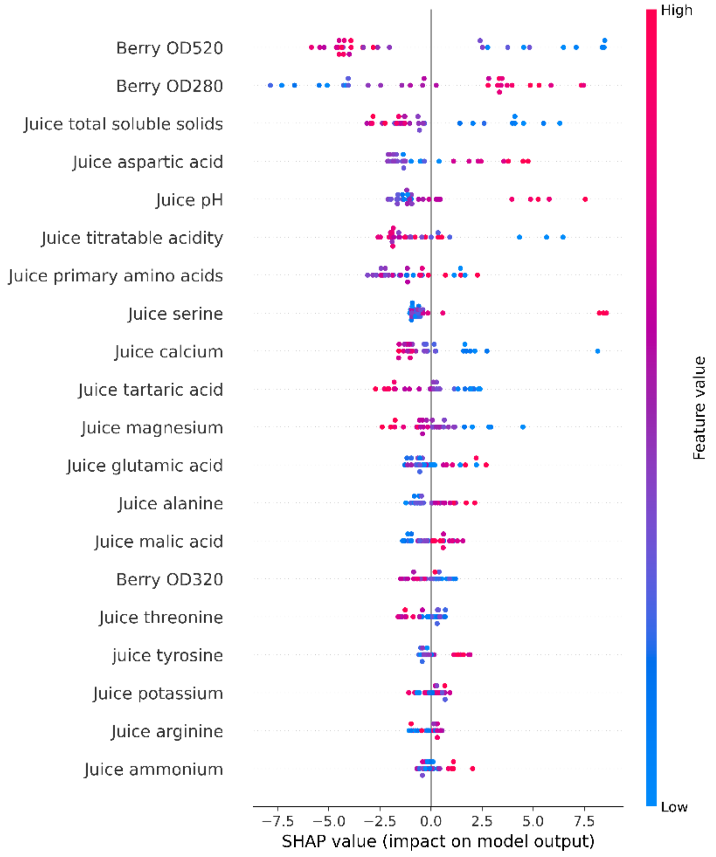

Figure S26: SHAP value summary plot for epicatechin levels in wine

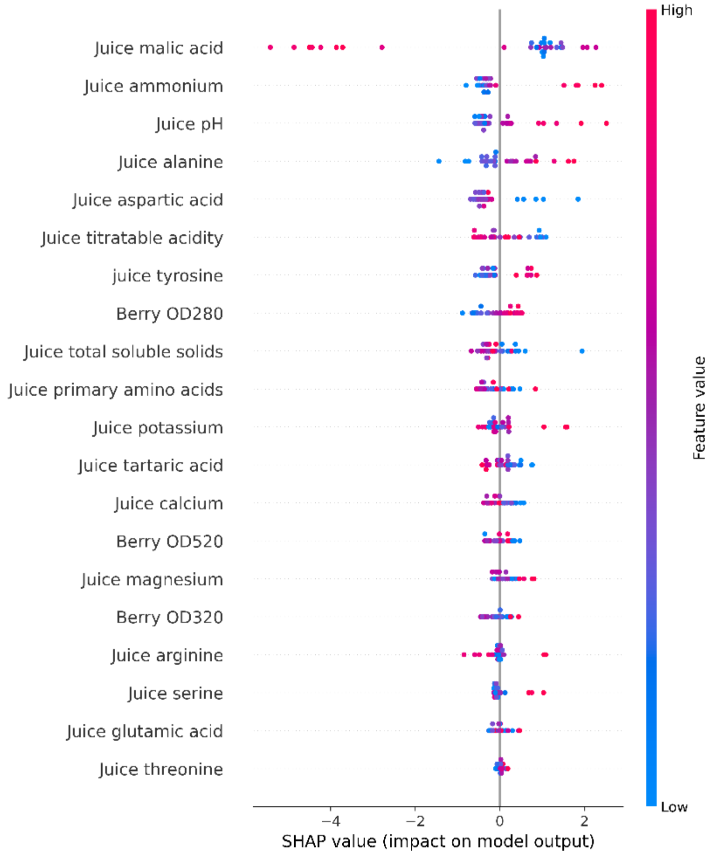

Figure S27: SHAP value summary plot for gallic acid levels in wine

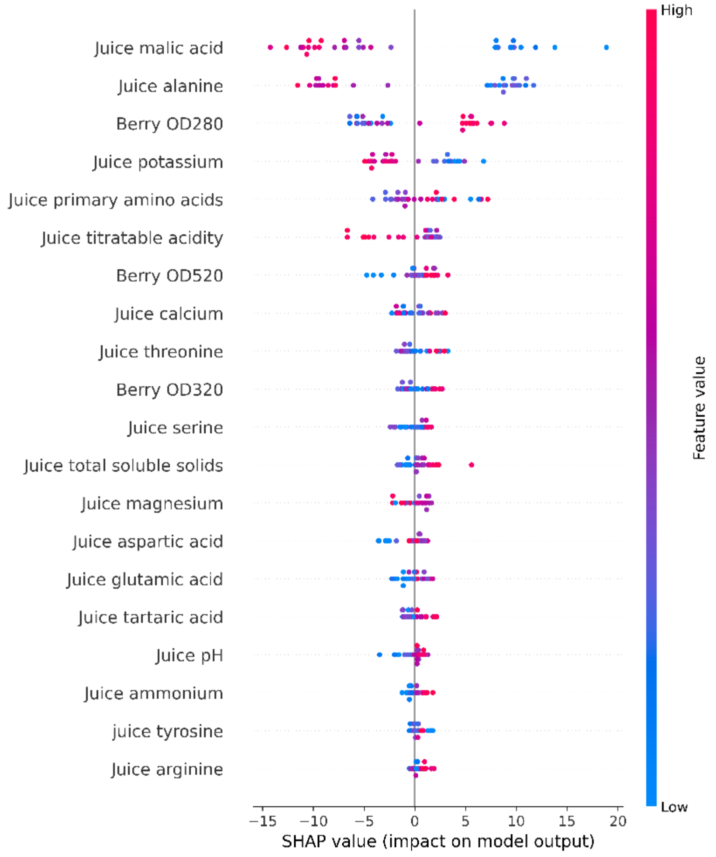

Figure S28: SHAP value summary plot for Malvidin 3-glucoside levels in wine

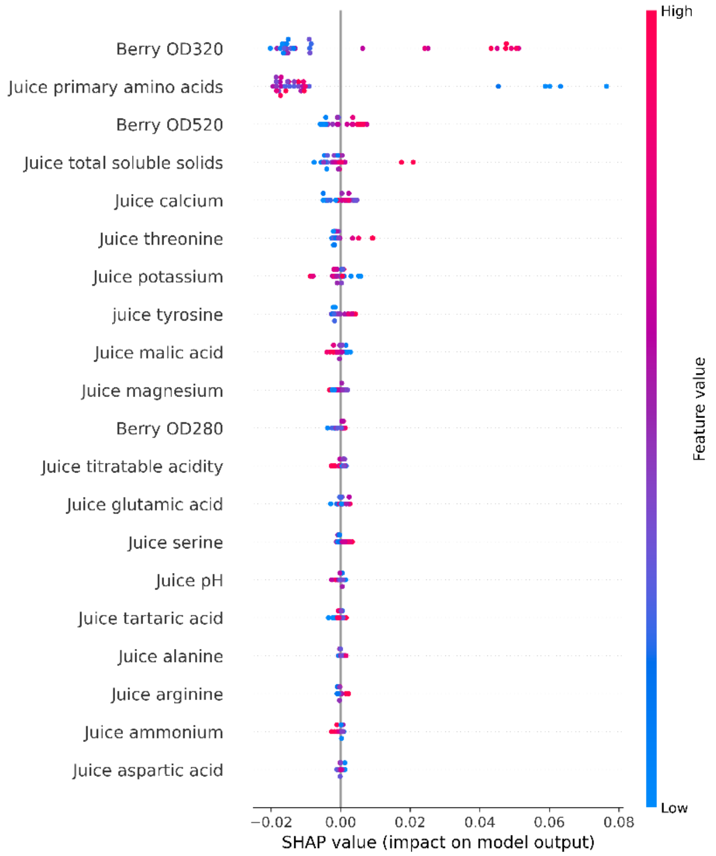

Figure S29: SHAP value summary plot for Mark to wine ratio in wine

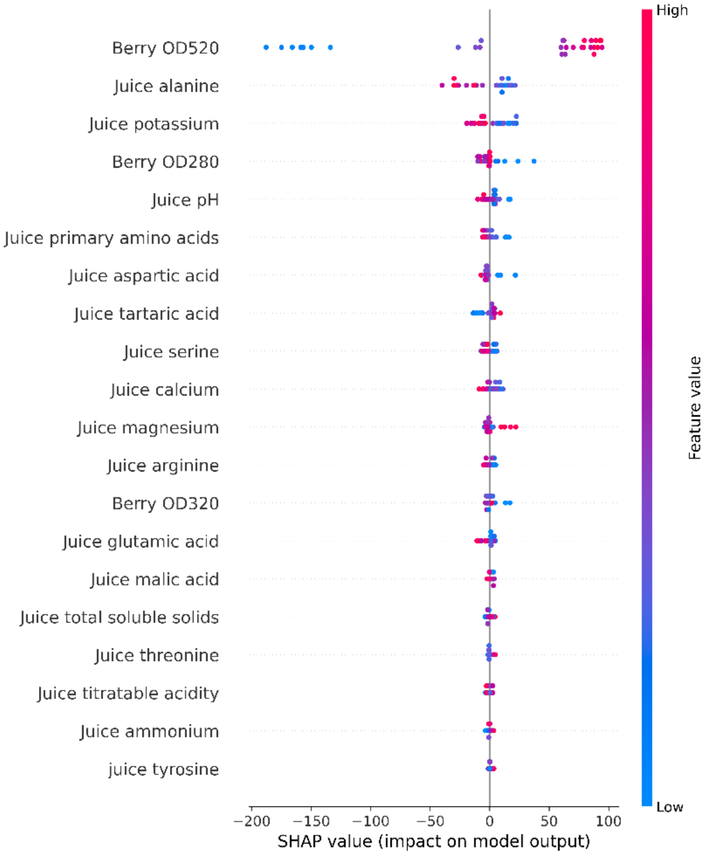

Figure S30: SHAP value summary plot for polymeric anthocyanin levels in wine

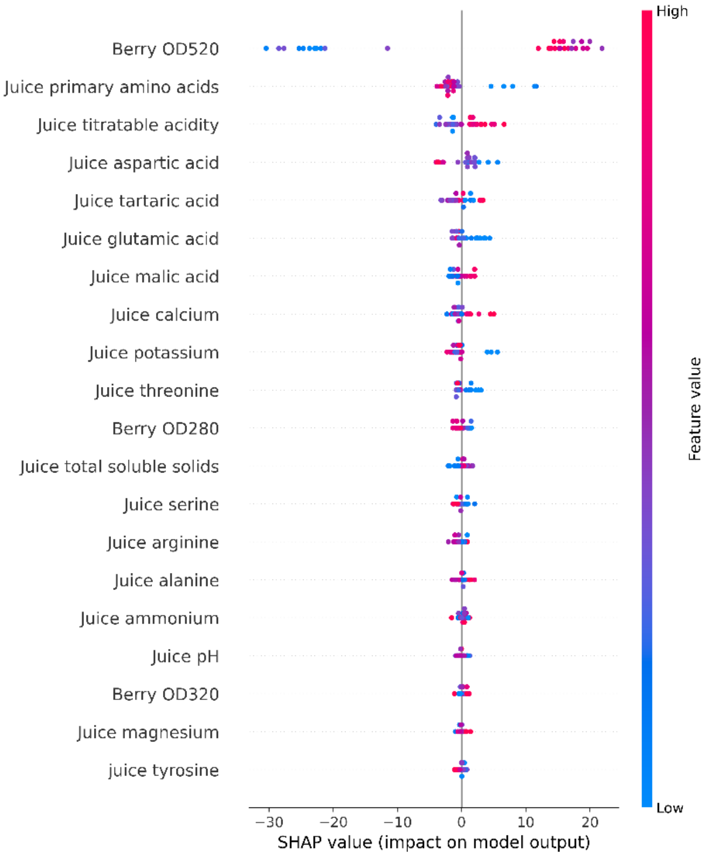

Figure S31: SHAP value summary plot for quercentin-G levels in wine

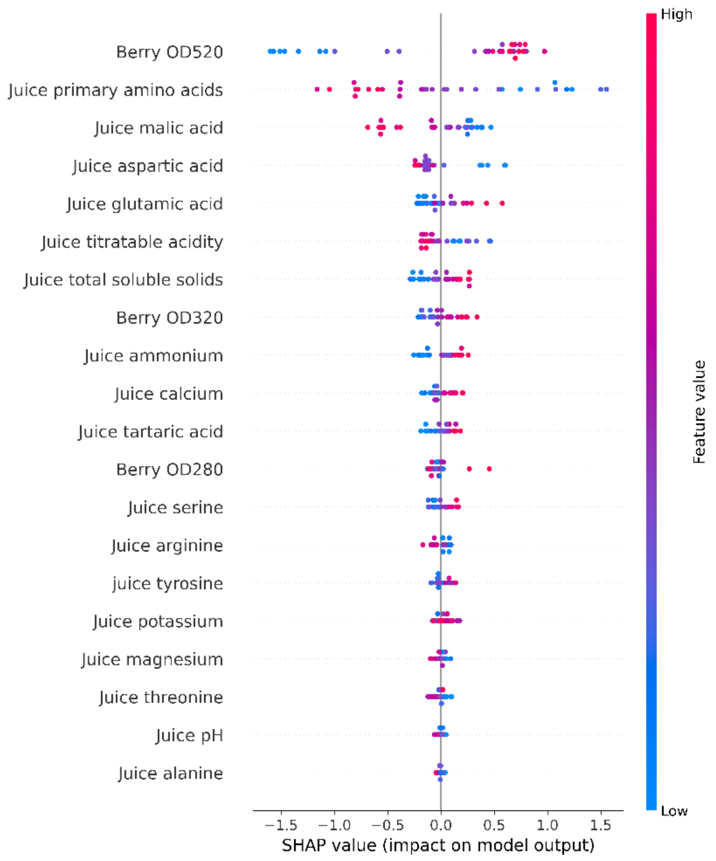

Figure S32: SHAP value summary plot for resveratrol levels in wine

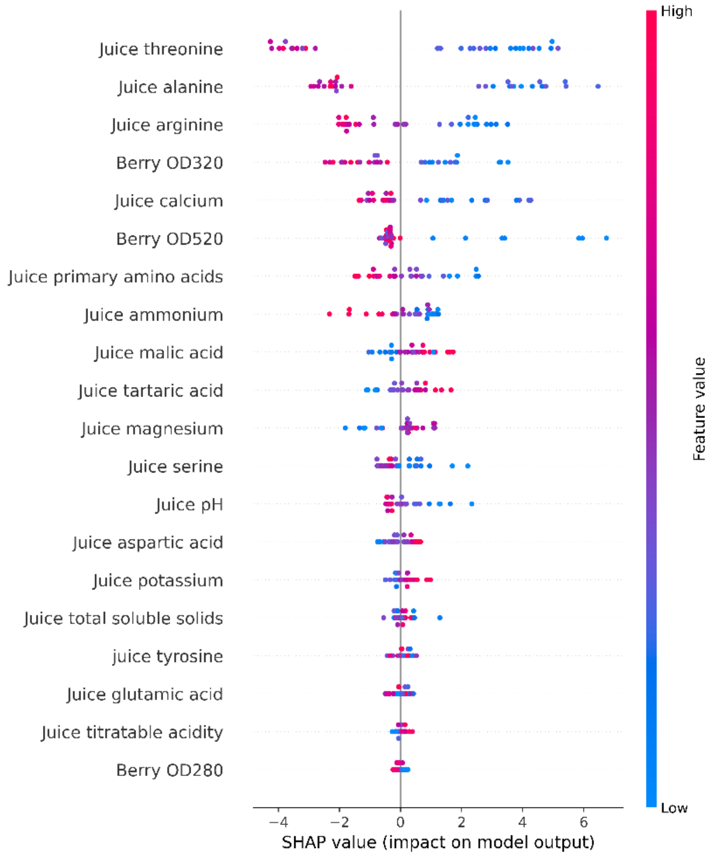

Figure S33: SHAP value summary plot for trans caftaric acid levels in wine

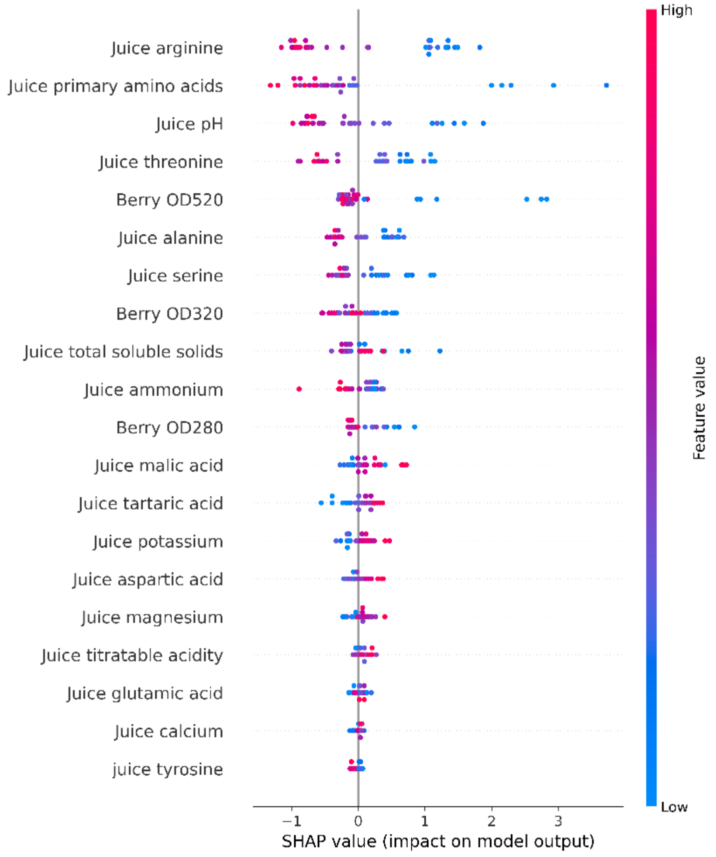

Figure S34: SHAP value summary plot for Trans-coutaric acid levels in wine

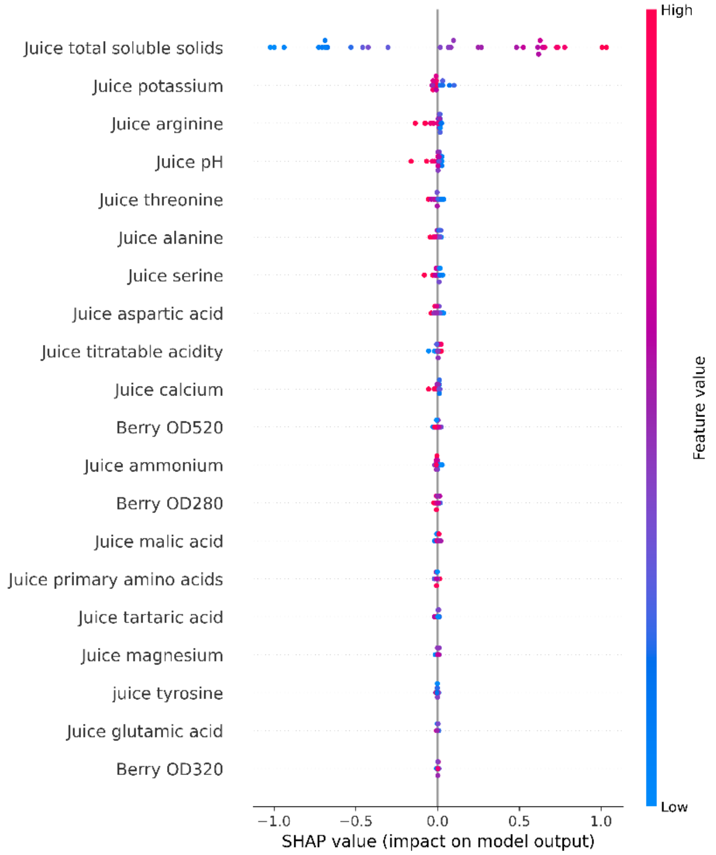

Figure S35: SHAP value summary plot for alcohol levels in wine

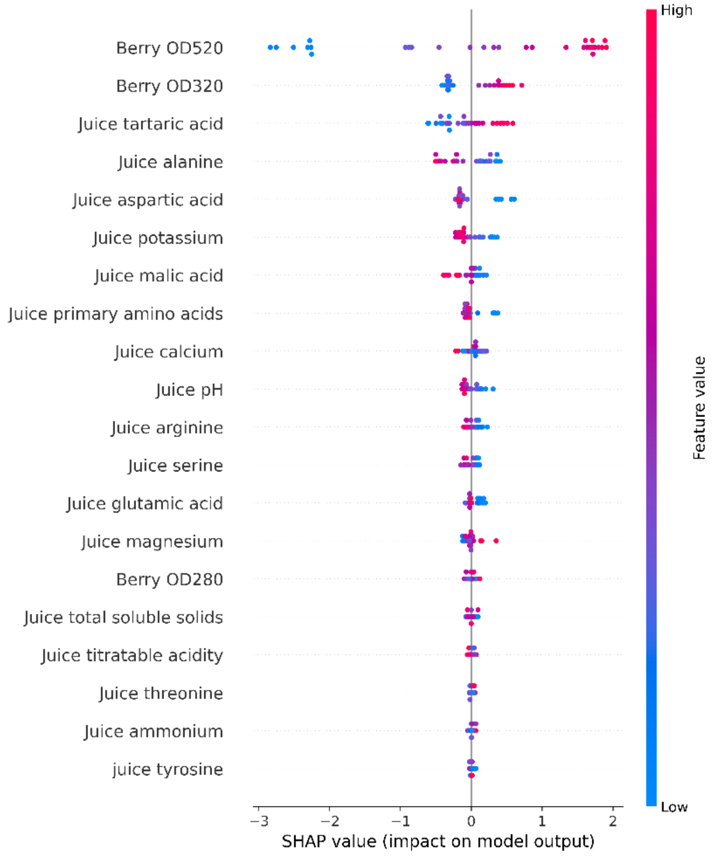

Figure S36: SHAP value summary plot for color density levels in wine

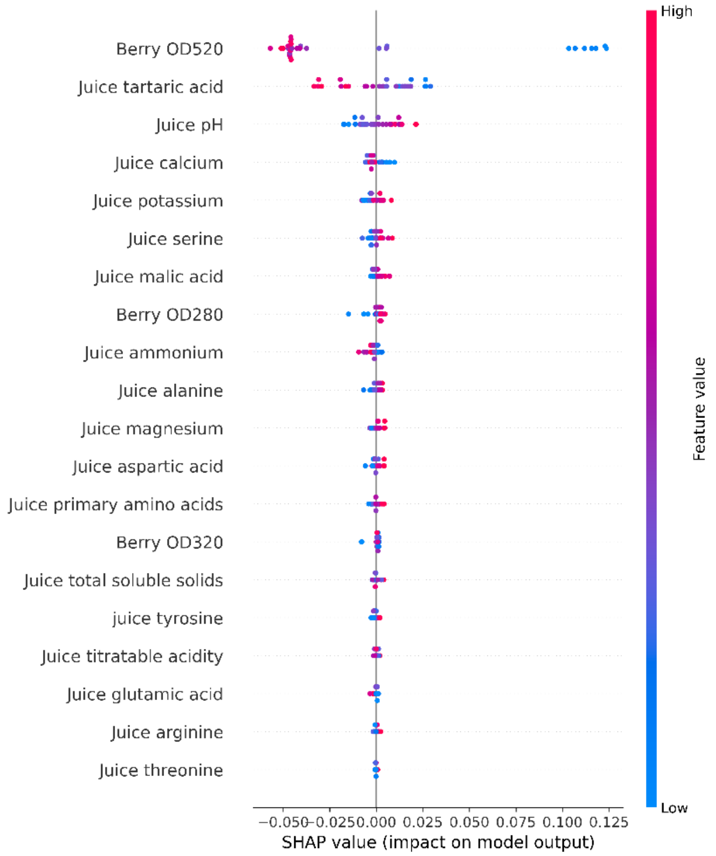

Figure S37: SHAP value summary plot for hue of wine

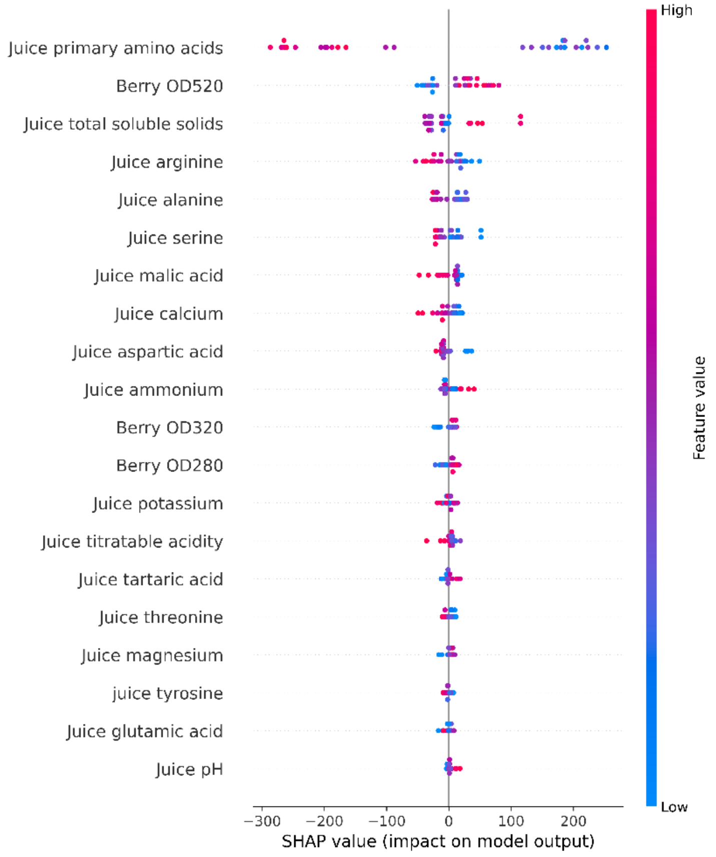

Figure S38: SHAP value summary plot for methyl cellulose precipitable tannins levels in wine

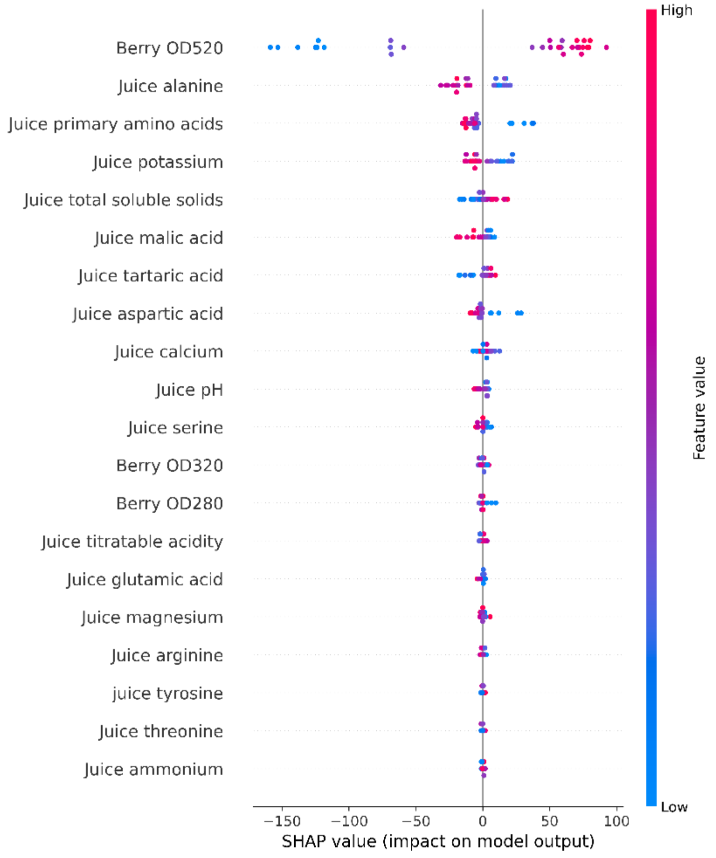

Figure S39: SHAP value summary plot for monomeric anthocyanin levels in wine

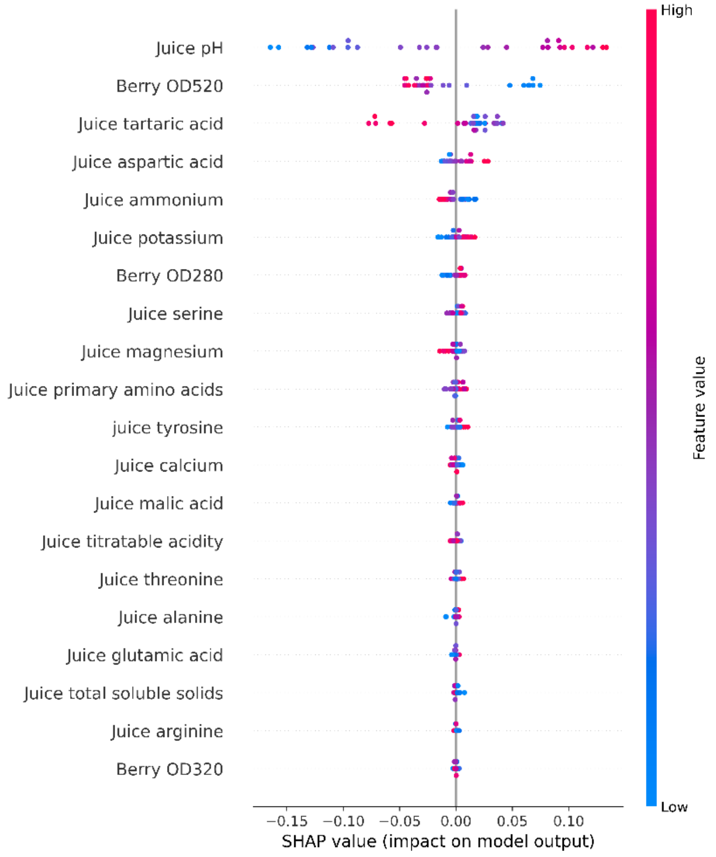

Figure S40: SHAP value summary plot for pH value in wine

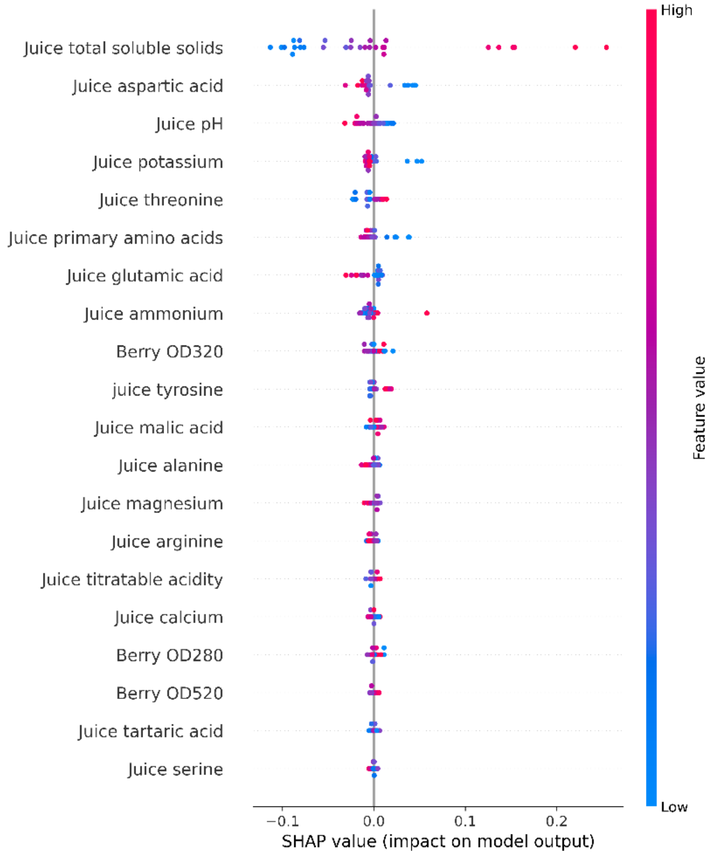

Figure S41: SHAP value summary plot for residual sugar levels in wine

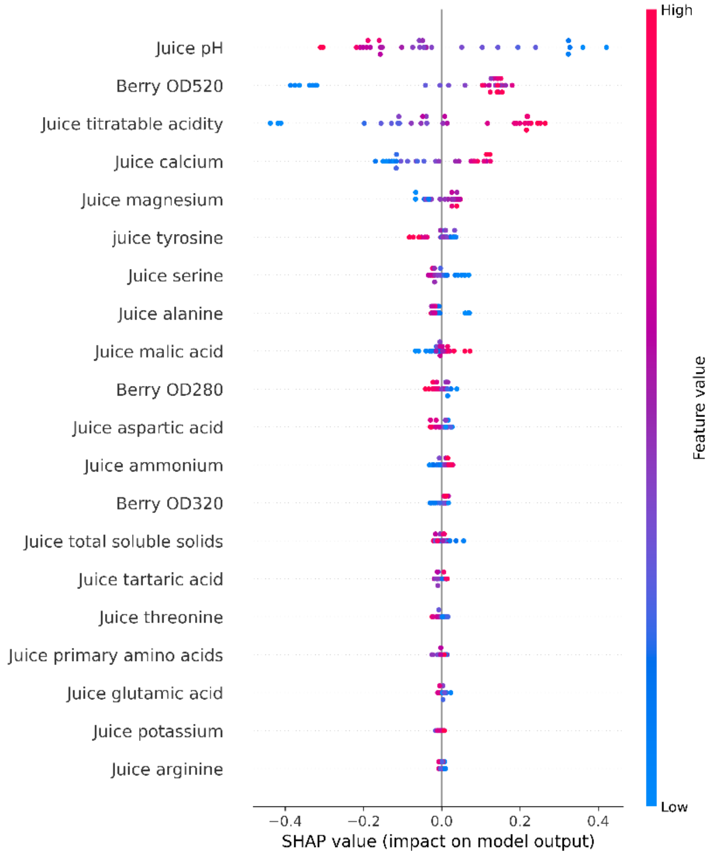

Figure S42: SHAP value summary plot for titratable acidity levels in wine

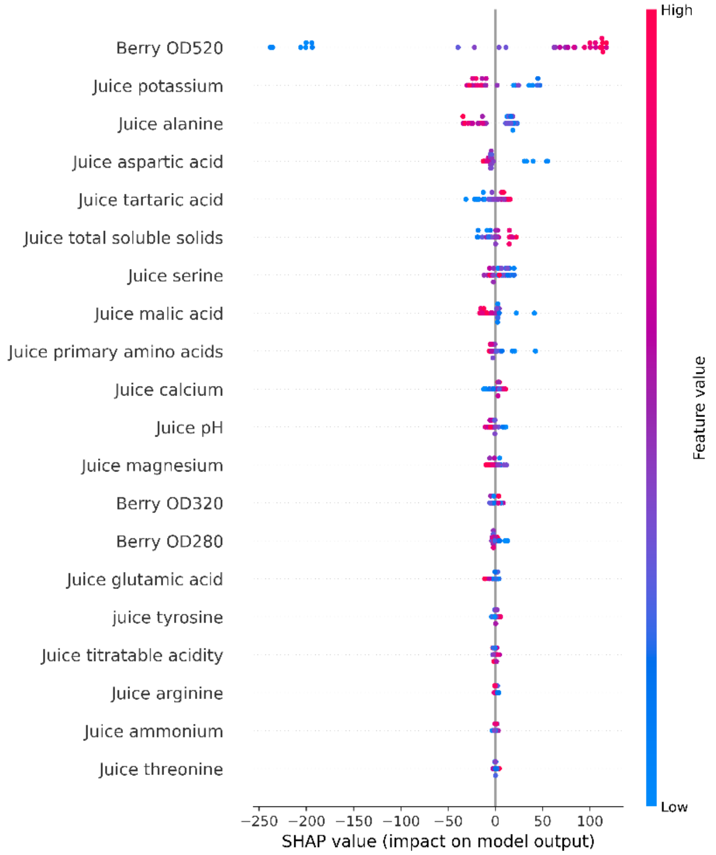

Figure S43: SHAP value summary plot for total anthocyanin levels in wine

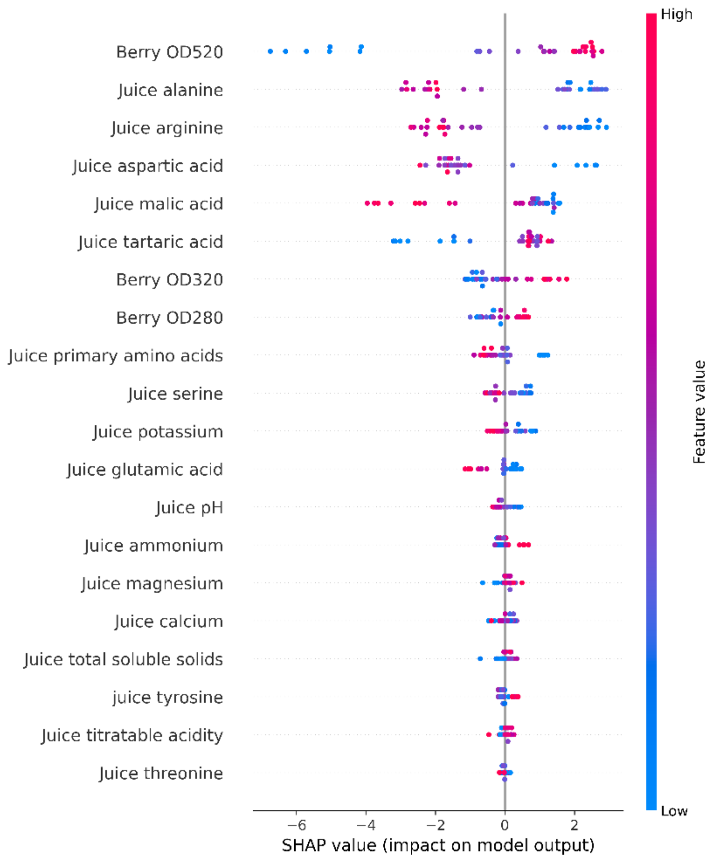

Figure S44: SHAP value summary plot for total phenolic levels in wine

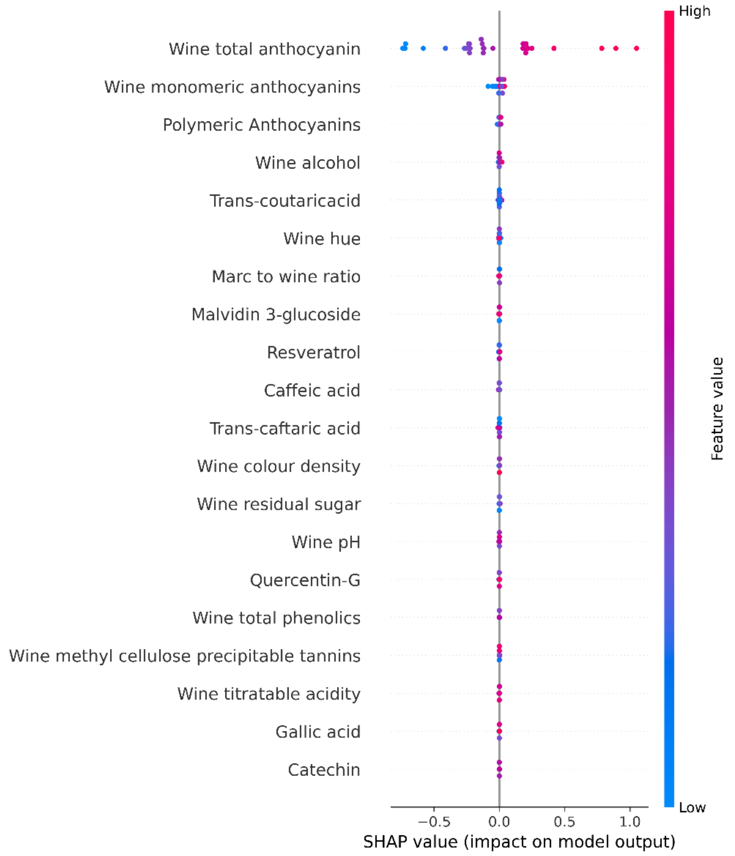

Figure S45: SHAP value summary plot for the quali

S2: Log normal transformations of inputs and exponential transformations for outputs of the four models

Model 1 Inputs:

$$x1 = \ln \frac{x1}{10} + 1$$

$$x2 = \ln \frac{x2}{50} + 1$$

$$x3 = \ln \frac{x3}{10} + 1$$

$$x4 = \ln \frac{x4}{10} + 5$$

Model 1 Outputs:

$$y1 = (e^{y1} - 2) * 10$$

$$y2 = (e^{y2} - 2) * 10$$

$$y3 = (e^{y3} - 2) * 10$$

Model 2 Inputs:

$$x1 = \ln \frac{x1}{10} + 1$$

$$x2 = \ln \frac{x2}{20} + 20$$

$$x3 = \ln \frac{x3}{10} + 1$$

$$x4 = \ln x4 + 2$$

$$x5 = \ln \frac{x5}{1000} + 10$$

$$x6 = \ln x6 + 2$$

Model 2 Outputs:

$$y1 = (e^{y1} - 1)$$

$$y2 = (e^{y2} - 1)$$

$$y3 = y3$$

$$y4 = e^{y4}$$

$$y5 = e^{y5}$$

$$y6 = (e^{y6}) * 100$$

$$y7 = (e^{y7} - 1) * 10$$

$$y8 = e^{y8}$$

$$y9 = e^{y9} * 10$$

$$y10 = e^{y10} + 6$$

$$y11 = (e^{y11} - 2) * 100$$

$$y12 = (e^{y12} - 2) * 1000$$

$$y13 = (e^{y13} - 2) * 100$$

$$y14 = e^{y14}$$

Model 3 Inputs:

$$x1 = \ln \frac{x1}{10} + 1$$

$$x2 = \ln x2 + 1$$

$$x3 = \ln x3 + 2$$

$$x4 = \ln \frac{x4}{10}$$

$$x5 = \ln x5$$

$$x6 = \ln \frac{x6}{100}$$

$$x7 = \ln x7 + 1$$

$$x8 = \ln x8$$

$$x9 = \ln \frac{x9}{100} + 1$$

$$x10 = \ln \frac{x10}{1000} + 1$$

$$x11 = \ln \frac{x11}{1000} + 1$$

$$x12 = \ln \frac{x12}{1000} + 2$$

$$x13 = \ln \frac{x13}{200} + 2$$

Model 3 Outputs:

$$y1 = e^{y1}$$

$$y2 = e^{y2}$$

$$y3 = e^{y3} * 4$$

$$y4 = (e^{y4} - 1) * 500$$

$$y5 = (e^{y5} - 1) * 20$$

Model 4 Inputs:

$$x1 = \ln \frac{x1}{10}$$

$$x2 = \ln x2$$

$$x3 = \ln \frac{x3}{100}$$

$$x4 = \ln \frac{x4}{100}$$

$$x5 = \ln \frac{x5}{10}$$

Model 4 Output:

$$y1 = e^{y1} + 1$$

USER MANUAL FOR THE WEB APPLICATION

OPEN ANY BROWSER:

GO TO THIS URL:

https://somin-s-wineprediction-app-ewbkrm.streamlitapp.com/

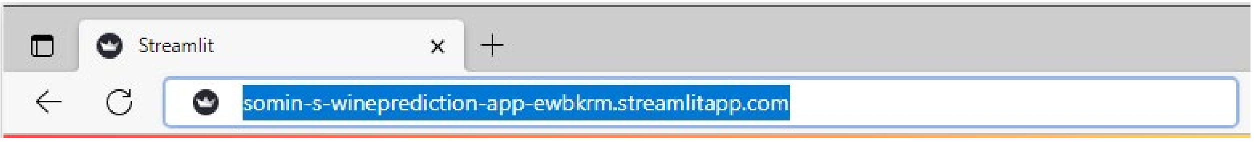

ARRANGE SIX INPUT PARAMETERS:

Users input six wine features via the side panels. Remark: System sets average values for the parameters as the default value.

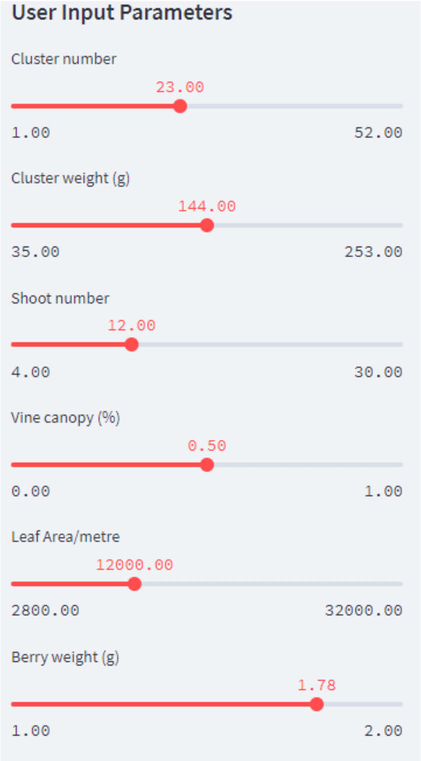

OUTPUT:

Three charts present predicted quality and value based on input wine features. The charts contain three types of data: yield per wine, yield per metre and yield per square metre in kilograms. The faceted chart separately presents the three types of data while the bigger chart present comparative information.

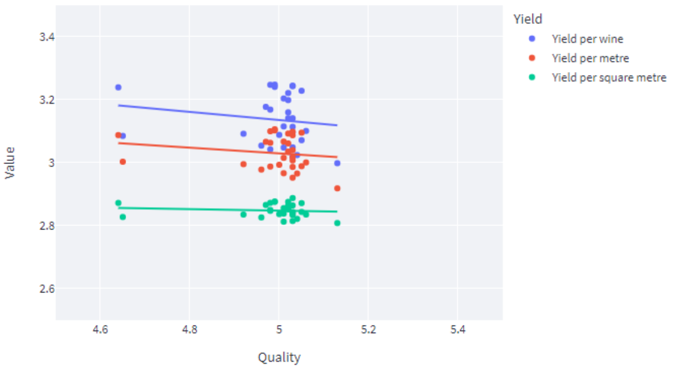

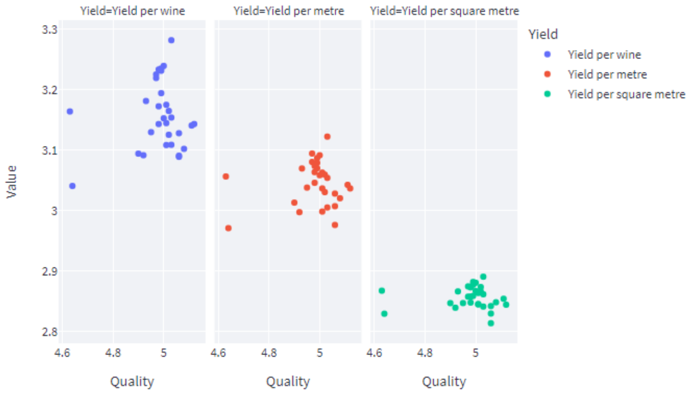

Click checkbox to show 20-sample data following the input parameters.

☑ Ouput 20 samples

	Berry OD280(AU)	Berry OD320(AU)	Berry OD520(AU)	Juice total soluble solids(oBrix)	Juice pH	Juice primary amino acids(g/L)	Juice malic acid(g/L)	Juice tartaric acid(g/L)
0	1.3184	0.2617	0.2540	21.9611	3.2806	216.9239	5.1890	3.9544
1	1.4018	0.2752	0.2742	21.8351	3.2720	218.5253	5.1442	4.0275
2	1.3929	0.2710	0.2644	21.8199	3.2787	214.8092	5.1312	4.0658
3	1.3006	0.2541	0.2426	22.4394	3.2521	214.0678	5.1491	4.1587
4	1.3147	0.2641	0.2590	22.1251	3.2721	217.7445	5.1404	4.0112
5	1.3764	0.2694	0.2686	22.0092	3.2691	219.8338	5.1477	4.0590
6	1.3021	0.2584	0.2559	22.0017	3.2847	214.7348	5.1616	3.9929
7	1.3814	0.2705	0.2628	21.8176	3.2656	217.5943	5.1458	4.0633
8	1.3598	0.2666	0.2574	21.7988	3.2279	218.2678	5.1722	4.2212
9	1.4116	0.2748	0.2715	22.0134	3.2477	218.7551	5.0856	4.1857
10	1.4160	0.2801	0.2784	21.6889	3.2883	215.7052	5.0839	4.1371
11	1.3430	0.2653	0.2501	21.8314	3.2381	217.2799	5.1638	4.2015
12	1.4193	0.2774	0.2652	21.7515	3.2285	218.2658	5.0947	4.3059
13	1.4025	0.2771	0.2749	21.7875	3.2818	216.6188	5.1231	4.0911
14	1.1885	0.1913	0.1752	22.3927	3.3689	221.5580	5.2572	3.9310
15	1.4192	0.2811	0.2790	21.8063	3.2648	217.2630	5.0710	4.1732
16	1.3376	0.2651	0.2619	21.8821	3.2752	216.5246	5.1787	3.9699
17	1.3953	0.2737	0.2630	21.7919	3.2300	218.7144	5.1081	4.2943
18	1.2777	0.2515	0.2476	22.0465	3.2192	218.5611	5.1869	4.2228
19	1.4792	0.2903	0.2717	21.7344	3.2189	219.9287	5.1103	4.2856

	Wine alcohol(% v/v)	Wine pH	Wine monomeric anthocyanins(mg/L M3G)	Wine total anthocyanin(mg/L M3G)	Wine total phenolics
0	13.5451	3.4698	333.9092	482.7299	50.4127
1	13.4056	3.4684	345.4286	496.8269	51.5717
2	13.4260	3.4637	340.7778	492.4622	51.2663
3	13.7999	3.4589	338.5350	492.8109	51.4227
4	13.5984	3.4624	349.4843	504.6987	52.0811
5	13.5714	3.4531	352.4795	511.0441	51.8254
6	13.5013	3.4703	332.1413	479.5097	50.2949
7	13.4701	3.4647	341.9725	492.9500	51.4042
8	13.5133	3.4553	335.4651	483.8804	51.5880
9	13.5762	3.4411	345.9668	505.5786	51.2061
10	13.3766	3.4631	340.6918	489.5483	51.1923
11	13.5604	3.4544	337.3585	490.9903	51.8046
12	13.5454	3.4344	337.6051	489.1238	51.2595
13	13.4036	3.4626	346.3673	500.0281	51.8242
14	13.7503	3.5760	285.9676	416.3141	44.9045
15	13.4179	3.4529	347.2314	498.5844	51.8304
16	13.5441	3.4669	346.4471	494.0307	51.9297
17	13.5310	3.4332	339.5400	498.3961	51.5502
18	13.6654	3.4384	345.0844	498.3127	51.6268
19	13.5599	3.4131	338.3288	492.9308	52.5172
